# Regulatory and disruptive variants in the *CLCN2* gene are associated with modified skin color pattern phenotypes in the corn snake

**DOI:** 10.1186/s13059-025-03539-0

**Published:** 2025-03-26

**Authors:** Sophie A. Montandon, Pierre Beaudier, Asier Ullate-Agote, Pierre-Yves Helleboid, Maya Kummrow, Sergi Roig-Puiggros, Denis Jabaudon, Leif Andersson, Michel C. Milinkovitch, Athanasia C. Tzika

**Affiliations:** 1 Laboratory of Artificial and Natural Evolution, Department of Genetics & Evolution, University of Geneva, Geneva, Switzerland; 2 Present address: Bracco Suisse S.A., Plan-les-Ouates, Switzerland; 3 Present address: Biomedical Engineering Program, Center for Applied Medical Research (CIMA), Universidad de Navarra, Instituto de Investigación Sanitaria de Navarra (IdiSNA), Pamplona, Spain; 4 Tierspital, University of Zurich, Zurich, Switzerland; 5 Department of Basic Neurosciences, University of Geneva, Geneva, Switzerland; 6 Clinic of Neurology, Geneva University Hospital, Geneva, Switzerland; 7 Department of Medical Biochemistry and Microbiology, Uppsala University, Uppsala, Sweden; 8 Department of Veterinary Integrative Biosciences, Texas A&M University, College Station, TX USA

**Keywords:** Reptiles, Skin, Coloration, Chromatophores, CLCN2

## Abstract

**Background:**

Snakes exhibit a broad variety of adaptive colors and color patterns, generated by the spatial arrangement of chromatophores, but little is known of the mechanisms responsible for these spectacular traits. Here, we investigate a mono-locus trait with two recessive alleles, *motley* and *stripe*, that both cause pattern aberrations in the corn snake.

**Results:**

We use mapping-by-sequencing to identify the genomic interval where the causal mutations reside. With our differential gene expression analyses, we find that *CLCN2* (Chloride Voltage-Gated Channel 2), a gene within the genomic interval, is significantly downregulated in Motley embryonic skin. Furthermore, we identify the *stripe* allele as the insertion of an LTR-retrotransposon in *CLCN2*, resulting in a disruptive mutation of the protein. We confirm the involvement of *CLCN2* in color pattern formation by producing knock-out snakes that present a phenotype similar to Stripe. In humans and mice, disruption of *CLCN2* results in leukoencephalopathy, as well as retinal and testes degeneration. Our single-cell transcriptomic analyses in snakes reveal that *CLCN2* is indeed expressed in chromatophores during embryogenesis and in the adult brain, but the behavior and fertility of Motley and Stripe corn snakes are not impacted.

**Conclusions:**

Our genomic, transcriptomic, and functional analyses identify a plasma membrane anion channel to be involved in color pattern development in snakes and show that an active LTR-retrotransposon might be a key driver of trait diversification in corn snakes.

**Supplementary Information:**

The online version contains supplementary material available at 10.1186/s13059-025-03539-0.

## Background

Animal color patterns play important roles in intra- and interspecific communication, such as species recognition, mate choice [1], and predator–prey interactions with camouflage and mimicry [2]. Variation in color patterns occurs within and among species, while convergence of adaptive patterns is sometimes found in distantly related lineages. As color patterns are under selective pressure, they provide excellent models to study the genetic and developmental basis of adaptive evolution. In vertebrates such as fish, amphibians, and reptiles, color patterns are the result of spatial arrangements of pigmentary and structural color cells called chromatophores. Melanophores and xanthophores contain black and yellow/red pigments, respectively, whereas arrays of guanine nanocrystals within iridophores produce a wide range of colors through light interference [3–5]. These cells originate in the neural crest during embryogenesis and migrate to the skin, where color patterns are generated via interactions among chromatophores and with their cellular environment [6–10].

The zebrafish is a primary model species for color pattern formation as more than 20 pattern-effecting loci have been identified (reviewed in ref. [11]). Some of the associated genes encode proteins involved in chromatophore formation and survival [12–15], but others affect the short-range interactions among pigment cells [16–19], such as gap and tight junction proteins (Connexin41.8, Connexin39.4, Tight junction protein 1a), proteins required for cell adhesion and migration (immunoglobulin superfamily member 11, Tetraspanin3c), and the inwardly-rectifying potassium channel Kcnj13. These data strongly suggest that cellular interactions, especially those based on direct cell–cell contacts among different chromatophore types, are crucial for color pattern formation in the zebrafish [7, 20, 21].

It remains to be investigated whether these mechanisms are conserved in other vertebrates, in particular in Squamates (lizards and snakes) that exhibit a remarkable set of pigmentary and structural elements generating a broad variety of adaptive colors and color patterns [4, 5, 22–26]. Spatial distribution of melanophores, xanthophores, and iridophores [4, 5, 22] in the skin of lizards and snakes generates patterns associated with habitat adaptation, reproductive strategies, aging, immune response, and speciation [27–29]. Different types of skin patterns in squamates have also been proposed to reflect different camouflage and behavioral strategies [1]: dorsal blotches are generally found in ambush hunting, and slow-moving snakes, such as pythons, while longitudinal stripes are often associated with the rapid escape speed of slender, fast-moving snakes [30, 31].

The corn snake (*Pantherophis guttatus*), a non-venomous, oviparous Colubridae species from the southeastern USA, is an ideal model for evolutionary developmental analyses of colors and color patterns in Squamates [32–37]. Indeed, as multiple color and pattern morphs have been isolated by private breeders in the last 65 years, one can contemplate the possibility of systematically analyzing the genetic basis of these traits and dissecting the corresponding molecular pathways controlling color pattern development and variation. With the availability of the necessary genomic [37, 38] and transcriptomic [39, 40] resources, it has been shown in corn snakes that (i) an LTR-retrotransposon insertion in *OCA2* (Oculocutaneous Albinism II) is responsible for the amelanistic phenotype [34], (ii) a disruptive mutation in *LYST* (lysosomal trafficking regulator) impacts the subcellular structure of all chromatophore vesicles, unifying them as lysosome-related organelles [37], and (iii) *PMEL* (premelanosome protein) is required for the differentiation and survival of all chromatophores during embryogenesis and its downregulation impacts the color patterning process [35]. Furthermore, a gene-editing protocol has been developed for corn snakes using CRISPR-Cas9 that allows to perform functional analyses [36].

**Fig. 1:**
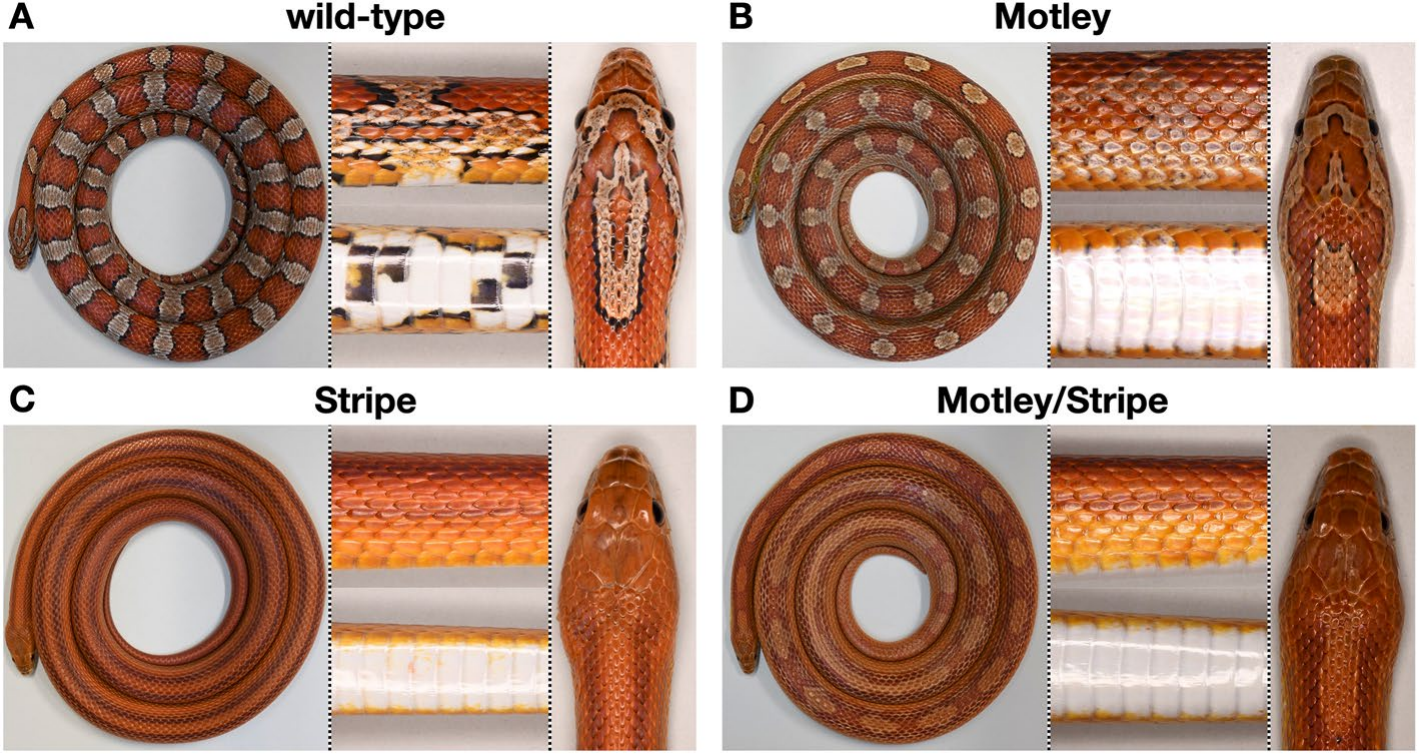
Color pattern of wild-type, Motley, Stripe, and Motley/Stripe corn snakes. **A** The wild-type pattern consists of dorsal and lateral blotches and black and white ventral checkers. **B** Motley individuals present elongated dorsal blotches that tend to fuse, especially at the anterior part of the body, with highly reduced lateral blotches and white ventral scales. **C** In Stripe individuals, the dorsal and lateral blotches are replaced by four stripes and the ventral scales are also white. Both the *motley* (*m*^*m*^) and *stripe* (*m*^*s*^) alleles are fully recessive to the wild-type allele: *m*^*m*^/ + and *m*^*s*^/ + individuals exhibit a wild-type phenotype as in **A**. **D** The phenotype of most *m*^*m*^/*m*^*s*^ animals is intermediate, combining fused blotches and stripes

Here, we focus on the Motley and Stripe traits first observed in the late 1970s, both of which present pattern aberrations (Fig. 1) and correspond to two alleles of the same locus, as supported by complementary crosses performed by corn snake breeders [30]. We combine mapping-by-sequencing [41] and transcriptomic analyses to identify the genomic interval where the causal mutations reside and to investigate the expression profile of the genes in the interval during development. Based on our findings, the Motley phenotype is associated with the downregulation of *CLCN2 *in embryonic skin, while a disruptive mutation affects the Stripe CLCN2 protein. We confirm the involvement of *CLCN2* in the coloration patterning process by generating knock-outs that resemble the Stripe animals. Further quantitative gene expression analyses, single-cell transcriptomic data, and histological examination confirm that *CLCN2* is expressed in the embryonic skin, the brain, and the retina of corn snakes. In Stripe individuals, we observe modifications in the skin coloration and vacuolation of the brain white matter due to the disruptive mutation. In Motley, the differential expression primarily impacts the coloration patterning. In conclusion, we determine that a plasma membrane anion channel protein plays a substantial role in the patterning process in the skin of corn snakes, underscoring the importance of cell-cell interactions in determining skin coloration and color pattern.

## Results

### The *motley* and *stripe* alleles modify the coloration pattern

The wild-type pattern of the corn snake (Fig. 1A) consists of dorsal and lateral red blotches on an orange background, whereas the ventral scales exhibit black and white checkers. The Motley phenotype (Fig. 1B) is characterized by elongated dorsal blotches that tend to fuse together at the edges and reduced and less well-defined lateral blotches than the wild-type. As its name implies, this phenotype is highly variable along the body of a single individual and among Motley individuals. Yet, they are all characterized by the absence of the ventral black checkers. In Stripe animals (Fig. 1C), the dorsal and lateral blotches are replaced by four continuous red stripes, two dorsally and two laterally, that run along the entire length of the body. Stripe individuals also have uniformly white ventral scales. The *motley* (*m*^*m*^) and *stripe* (*m*^*s*^) alleles are recessive to the wild-type one. Heterozygous Motley/Stripe individuals (*m*^*m*^/*m*^*s*^) have a phenotype intermediate between Motley and Stripe with dorsal blotches that tend to fuse and occasionally form stripes. A great variation can be seen along the body of each heterozygous individual, as well as among individuals (Fig. 1D, Additional file 1: Fig. S1) [42].

### Identification of the genomic region bearing the *motley/stripe* locus

**Fig. 2:**
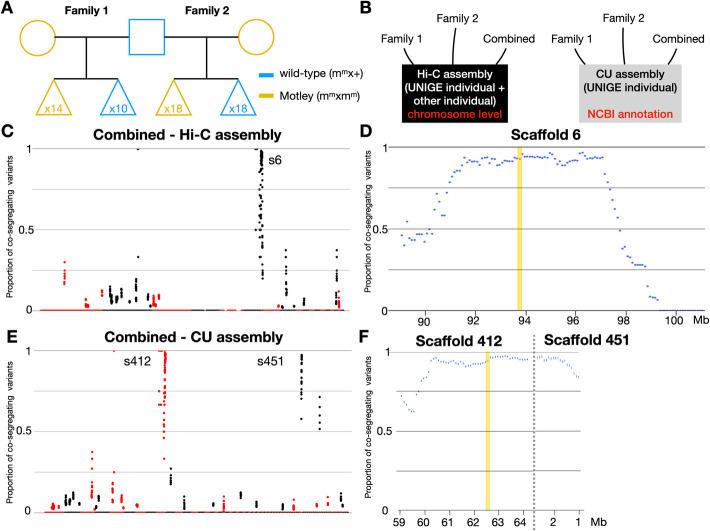
Mapping of the Motley variant.** A** Pedigree of the two families that were used separately and combined for the mapping of the *motley* allele. No mapping was performed for the *stripe* allele, because it corresponds to the same locus as the *motley* allele. **B** Each family separately and the two families combined were mapped both to the Hi-C and the CU assembly.** C** Proportion of co-segregating variants with the Motley locus (*y*-axis) in the two families combined when mapped to the Hi-C assembly. Proportions are calculated with a 1-Mb sliding window and a step of 100 kb. Scaffolds are alternatively colored in red and black. **D** Proportion of co-segregating variants in the interval between 91.4 and 97.1 Mb on Scaffold 6 (s6) of the Hi-C assembly based on the combined dataset. Proportions are calculated with a 1-Mb sliding window and a step of 100 Kb. The position of the main candidate gene (*CLCN2*) is highlighted in yellow.** E** Proportion of co-segregating variants with the Motley locus (*y*-axis) in the two families combined when mapped to the CU assembly. Proportions are calculated with a 1-Mb sliding window and a step of 100 kb. Scaffolds are alternatively colored in red and black. **F** Proportion of co-segregating variants in the 3.4 Mb interval on Super-scaffolds 412 (61.6–64.1 Mb) and 451 (reverse complement from 2 to 1.1 Mb) of the CU assembly based on the combined dataset. Proportions are calculated with a 1-Mb sliding window and a step of 100 Kb. The position of the main candidate gene (*CLCN2*) is highlighted in yellow

Given that the *motley* and *stripe* alleles reside on the same locus, we performed mapping-by-sequencing as previously described [37] only for the *motley* allele. No mapping was done for the *stripe* allele. We used whole-genome Illumina sequencing of two families, each consisting of four libraries for (i) a heterozygous wild-type male (*m*^*m*^/ +), (ii) a homozygous Motley female (*m*^*m*^/*m*^*m*^), (iii) a pool of heterozygous offspring (*m*^*m*^/ + ; 14 in family 1 and 18 in family 2), and (iv) a pool of homozygous offspring (*m*^*m*^/*m*^*m*^; 10 in family 1 and 18 in family 2) (Table S1; NCBI accession PRJNA1143197). The same male was used for both family crosses, but we generated two libraries for this individual (Fig. 2A). We aligned each library separately to the Hi-C chromosome-level re-assembly of the corn snake genome from DNA Zoo [43, 44] and to the CU assembly [45]. We performed the mapping on both assemblies to benefit from (i) the continuity of the chromosome-level DNA Zoo assembly and (ii) the high-quality NCBI annotation of the CU assembly (Fig. 2B). We searched for indels, single- and multi-nucleotide polymorphisms co-segregating with the Motley genotype in non-repetitive elements [37].

Based on the Hi-C assembly, the results of the mapping for the two families are slightly different (Additional File 1: Fig. S2A, B), reflecting the differences in (i) the genetic background of the two females, as the male was the same and (ii) the sequencing depth, which was greater for family 1 (38–75 ×) compared to family 2 (12–21 ×) (Additional File 1: Table S1). In family 1, we observe a high variant co-segregation (up to 82%) in an interval of 3.2 Mb on Scaffold 6 (92.1–95.3 Mb; Additional File: Fig. S2C). A 50 Mb region of lower co-segregation (maximum 64%) is also evident on Scaffold 2 (Additional File 1: Fig. S2A). In family 2, we find the same interval on Scaffold 6 with 99% co-segregation but extended to 6.5 Mb (90.9–97.4 Mb; Additional File 1: Fig. S2B, D). Secondary peaks with lower co-segregation are also seen on Scaffolds 1, 2, 8, and 14 (Additional File 1: Fig. S2B). When the libraries of the two families are combined, a single 5.7 Mb interval on Scaffold 6 dominates (91.4–97.1 Mb), with 345 co-segregating variants (61 variants/Mb) (Fig. 2C, D). We obtain similar results with the mapping on the more fragmented CU assembly for each family separately (Additional File 1: Fig. S2E, F) and when combining them (Fig. 2E). The exact same interval on Scaffold 6 of the Hi-C assembly is split in Super-scaffolds 412 (NW_026844092.1—60.1–64.1 Mb) and 451 (NW_026844135.1—reverse complement from the end of the scaffold at 2.8 Mb to 1.1 Mb; Fig. 2F). The presence of additional intervals with relatively high percentage of co-segregating variants in our mapping suggests that the parents are likely carriers of additional mutations that partially co-segregate in the families we sequenced. We performed the mapping on two families to exclude these by-chance co-segregating intervals. The combined results strongly support that the *motley* allele resides on Scaffold 6.

To reduce the genomic interval, we genotyped by Sanger sequencing the 22 offspring of family 1, as well as 10 additional *m*^*m*^*/* + and *m*^*m*^*/m*^*m*^ offspring from another cross of the same male with a different female (Additional file 1: Fig. S3). More specifically, we amplified and sequenced short fragments (200–800 bp) within the interval and looked for recombination events at predefined sites where fixed mutations are expected. The sites were selected based on our mapping-by-sequencing analyses. We first verified their state (homozygous/heterozygous) in the parents of the genotyped offspring. For example, we would expect a phenotypically Motley individual to be homozygous at these sites. If a recombination occurred, it would instead be heterozygous at the same sites. We identified five recombinant individuals and thus reduced the interval to 3.4 Mb on Super-scaffolds 412 (61.6–64.1 Mb) and 451 (reverse complement from 2 to 1.1 Mb). This corresponds to a 3 Mb interval on Scaffold 6 (92.9–95.9 Mb). The difference in the length between the two assemblies is due to a 370 Kb stretch of unidentified bases (Ns) in the Hi-C assembly, corresponding to the junction of the two CU assembly scaffolds.

We then proceeded with the variant calling on the CU assembly, for which a high-quality NCBI annotation is available. Among the 48 coding genes in the reduced interval, 33 carry indels, single- and multi-nucleotide polymorphisms that co-segregate with the Motley genotype, most of which are in untranslated regions (Additional File 1: Table S2). Although no high-impact variants were detected, such as the introduction of stop codons, deletions/insertions of amino acids, and mutations on intron donor and acceptor sites, four genes (*VWA5B2*, two *cytochrome P450-like* genes, and *LOC132709391*, an uncharacterized gene encoding a long-non coding RNA) carry mutations that result in the non-synonymous substitution of one or two amino acids. These missense mutations have no impact neither on the domain prediction of the proteins according to InterProScan [46] nor on their three-dimensional structure according to SWISS-MODEL [47]. No protein sequence is available for *LOC132709391*. In conclusion, we could not detect any disruptive mutations responsible for the Motley phenotype within the genomic interval.

### Downregulation of *CLCN2* in Motley corn snakes

**Fig. 3:**
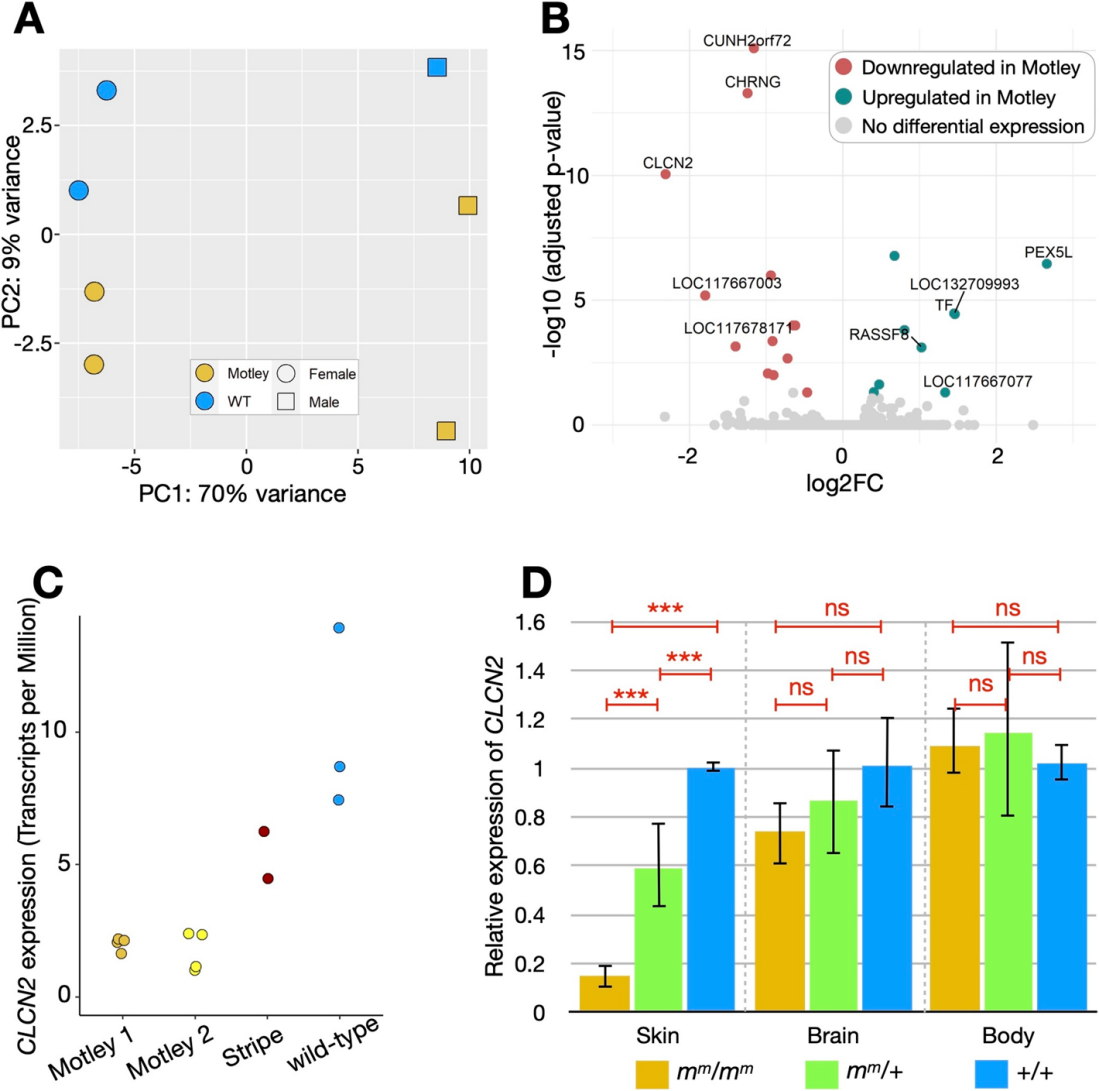
Bulk RNA-seq differential expression analyses and quantitative PCR on embryonic tissues. **A** Principal component analysis of the seven RNA-seq samples that primarily cluster based on their sex at this early stage of development when pigmentation is not visible yet. **B** Volcano plot depicting statistically significant gene expression changes between wild-type and Motley embryonic dorsal skin samples in terms of log2 fold-change (*x*-axis) and negative log10 of adjusted *p*-value (*y*-axis). In cyan, the genes significantly upregulated in Motley, and in red, the ones significantly downregulated (cutoff adjusted *p*-value: 0.05). The ten genes with a log2 fold-change > 1 are labeled. *LOC117667003* is ryncolin-3, *LOC117678171* is calcium-activated potassium channel subunit beta-2 (*KCNMB2*), *LOC132709993* is an uncharacterized protein C2orf72-like, and *LOC117667077* is an uncharacterized protein. **C** Expression levels of *CLCN2* in Transcripts per Million in Motley, Stripe, and wild-type samples. Despite that the samples are from two different clutches (Motley 1 with wild-type and Motley 2 with Stripe), we observe similar levels of expression for all Motley samples from the two crosses. The expression in Stripe is similar to the wild-type expression. **D** Gene expression ratio of *CLCN2* in the embryonic skin, the brain, and a body part compared to the wild-type for three genotypes. The *CLCN2* expression is significantly downregulated in the Motley embryonic skin sample, compared to the heterozygous and the wild-type, based on a Welch’s *t*-test. The three asterisks denote a *p*-value < 0.001 and “ns” a *p-*value > 0.05. The error bars correspond to the standard deviations

To verify whether the *motley* allele corresponds to a regulatory mutation that impacts the expression levels of one of the genes in the genomic interval, we performed differential expression analyses by bulk RNA sequencing (NCBI accession GSE273807). We extracted RNA from the skin of four homozygous Motley (*m*^*m*^*/m*^*m*^) and three wild-type (+ */* +) embryos at the developmental stage 7 (embryonic day 20). We have previously shown that unpigmented chromatophore progenitors are already present in the skin at this stage and the patterning process has been initiated [35]. All embryos were from the same cross (*m*^*m*^*/* + × *m*^*m*^*/* +) and clutch to minimize expression differences due to a variable genetic background and developmental stage. The samples tend to primarily cluster based on their sex (Principal Component 1—PC1: 70%) and less so on their phenotype (PC2: 9%), which is expected given that the pigmentation process is only at its early stages (Fig. 3A). We observed the same result (PC1 distinguishing sex and PC2 distinguishing color pattern phenotype) when analyzing embryonic skin RNA-seq samples of a different corn snake morph at the same developmental stage [35]. Only 22 genes were significantly differentially expressed (adjusted *p*-value < 0.05) between the two conditions (Fig. 3B; Additional File 2), with ten of them presenting an absolute log2 fold-change greater than 1. *PEX5L* (peroxisomal biogenesis factor 5-like) is the most upregulated gene (adjusted *p*-value: 3.43.10^−7^; log2 fold-change: 2.66), and *CLCN2* (Chloride Voltage-Gated Channel 2) is the most significantly downregulated gene (adjusted *p*-value: 8.88.10^−11^; log2 fold-change: − 2.31). Among the 22 differentially-expressed genes, only *CLCN2* is within the reduced interval. Eleven out of the 21 remaining differentially-expressed genes are located within 5 Mb of either side of the reduced interval, suggesting that their expression might be affected by the same regulatory mutation. We confirmed the expression of *CLCN2* in chromatophore progenitors, thanks to the single-cell transcriptomic analyses we previously performed on the skin of a wild-type corn snake embryo at a similar developmental stage [35] (Additional File 2).

We also compared the changes in gene expression levels between two Stripe and four Motley stage 7 embryos that were produced from a *m*^*m*^*/m*^*s*^ × *m*^*m*^*/m*^*s*^ cross. *CLCN2* is among the 21 differentially expressed genes in this experiment, as well as a *cytochrome P450-like* gene from the reduced interval (Additional File 2). We observe again low levels of *CLCN2* expression in the Motley samples, whereas the expression of one of the Stripe samples is similar to the expression of one of the wild-type samples (Fig. 3C).

To test our bulk RNA-seq findings for *CLCN2*, we performed real-time quantitative PCR on three types of tissue at the same developmental stage. We sampled the skin, the brain, and a body piece consisting of connective tissue, developing muscles and bones, but no skin and no internal organs. We analyzed four *m*^*m*^*/m*^*m*^, eight *m*^*m*^*/* + , and two + / + individuals with primers spanning *CLCN2* exons 17 and 18 (Fig. 3D). Only one *CLCN2* isoform is annotated in the corn snake CU genome, but up to three isoforms have been annotated in other snakes, such as the Tiger rattle snake (*Crotalus tigris*—NCBI Gene ID 120313961) and the viper boa (*Candoia aspera*—NCBI Gene ID 134499625). All isoforms in these species maintain exons 17 and 18. We were able to confirm a significant downregulation of *CLCN2* in the skin of the homozygous animals. In the brain, the average expression level is lower in homozygous Motley animals compared to heterozygous and wild-type, but the differences are not statistically significant. Similar levels of expression are observed in all samples of the body part. We searched for fixed mutations in the promoter region of *CLCN2*—within 1000 bases upstream of the 5′ UTR—but none was found in the Motley gDNA libraries that we sequenced for the mapping. Overall, we hypothesize that the regulatory mutation that results in the Motley phenotype specifically affects the skin.

### An LTR-retrotransposon insertion in the Stripe *CLCN2*

**Fig. 4:**
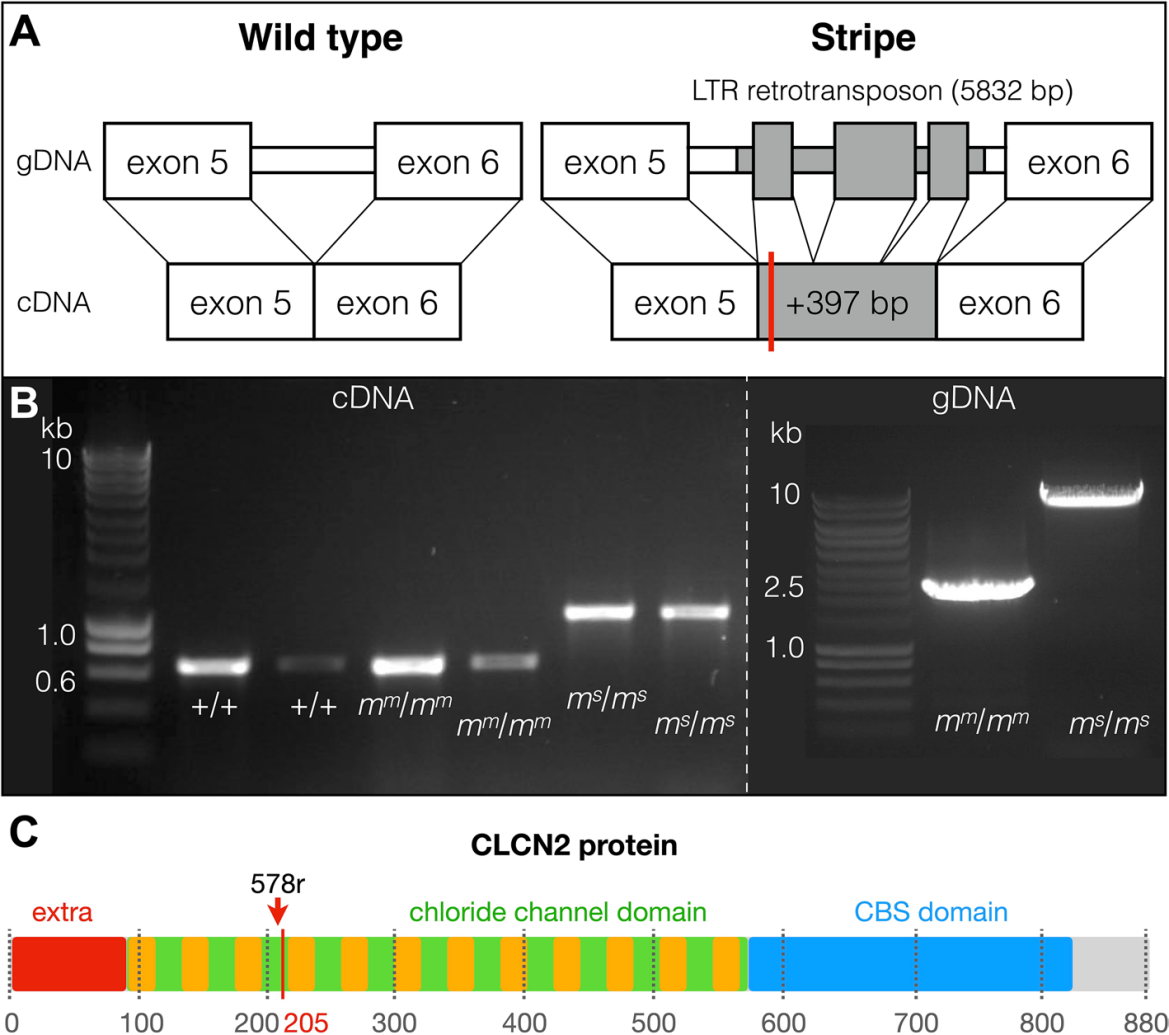
An LTR-retrotransposon insertion in the Stripe *CLCN2*. **A** The *stripe* mutation corresponds to the insertion of a 5832-bp LTR-retrotransposon (gray) in the fifth intron of the *CLCN2* gene. Three fragments (not drawn to scale) of the retrotransposon element are spliced together and a 397-bp sequence is inserted between exons 5 and 6 of the *CLCN2* Stripe transcript. As a result, four amino acids are introduced after exon 5 followed by a premature stop codon (red line). **B** Agarose gel images of PCR products of the *CLCN2* fragments from cDNA (left) and gDNA (right) of wild-type (+ / +), Motley (*m*^*m*^/*m*^*m*^), and Stripe (*m*^*s*^/*m*^*s*^) individuals. The 397-bp insertion is visible in the cDNA of the Stripe samples and the 5832-bp insertion in the gDNA of the Stripe samples. **C** Graphical representation of the domains of the corn snake CLCN2 protein. The premature stop codon is at position 205 and the red arrow points to the location of the gRNA used for gene-editing. Extra: extracellular domain, orange rectangles: transmembrane domains (not drawn to scale)

Given that the *motley* and *stripe* alleles reside on the same locus, and *CLCN2* is not differentially expressed in the skin of Stripe individuals, we investigated for the presence of disruptive mutations. By Sanger sequencing the *CLCN2* transcript and the corresponding genomic region from *m*^*m*^*/m*^*m*^, *m*^*s*^*/m*^*s*^, and + / + animals, we identified an insertion in the *CLCN2* gene of Stripe corn snakes (Fig. 4A, B; Additional file 3). As the NCBI annotation of *CLCN2* is not complete, we manually curated it by replacing a stretch of Ns in the CU assembly with the corresponding sequence from the Hi-C assembly (Additional files 4 and 5). The wild-type *CLCN2* contains 24 exons generating a 2643 bp coding sequence and an 880 aa protein. The *CLCN2* Stripe transcript includes a 397 bp insertion, between exons 5 and 6, that introduces a premature stop codon and truncates the protein to only 205 aa (Additional file 3). As a result, the chloride channel core domain is shortened from twelve to two transmembrane regions and the cystathionine-beta-synthase (CBS) domain is absent (Fig. 4C; Additional file 1: Fig. S4) based on the predictions by InterProScan and PROTTER [48]. In the bulk RNA sequencing libraries of Stripe embryos, we identified three reads spanning the insertion and the upstream/downstream regions (Additional file 1: Fig. S5). No such reads were found in the Motley and wild-type samples.

**Fig. 5:**
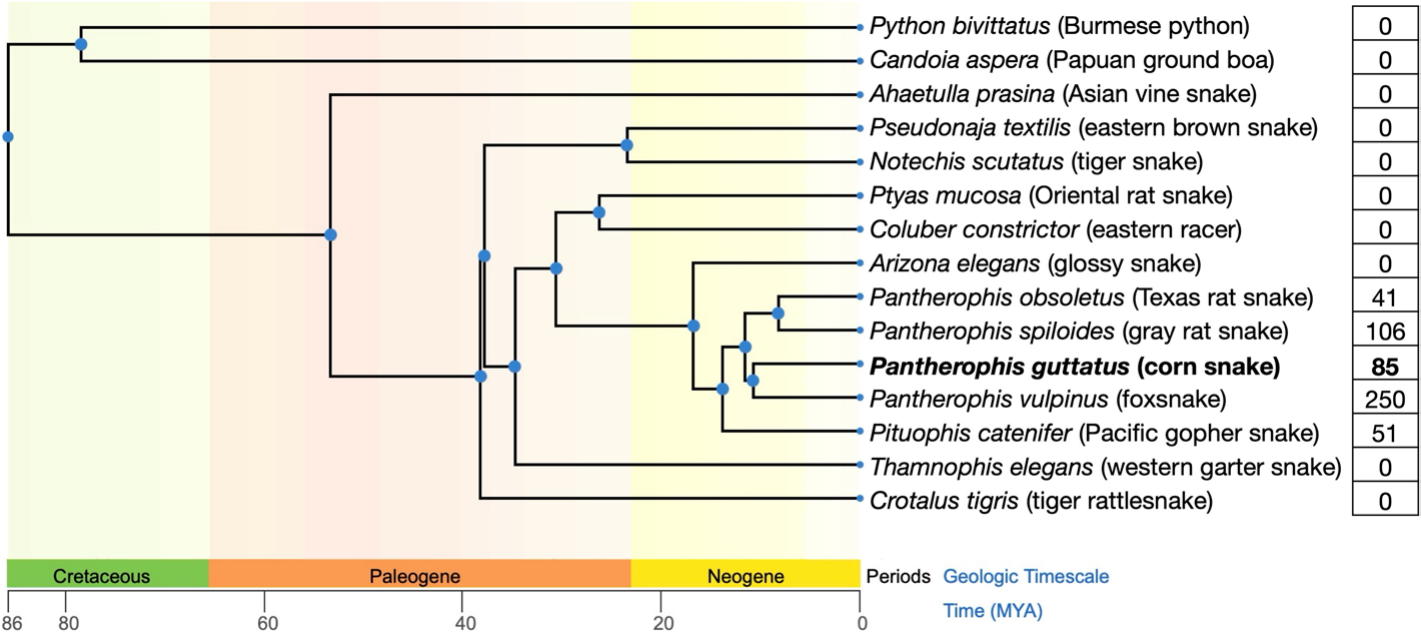
Presence of the Copia LTR-retrotransposon in different snake lineages. Phylogenetic tree incorporating the interrogated snake genomes. The branch lengths correspond to estimated divergence in MYA [49]. The numbers on the right correspond to the number of the Copia LTR-retrotransposon copies found in each genome. Depending on the continuity of the genomes, some copies might have been missed. The corn snake is highlighted in bold and the common species names are given in parenthesis. The tree was built using TimeTree v. 5 [49]

The insertion in the Stripe *CLCN2* resembles in length and sequence the insertion in the *OCA2* gene that we previously identified as the causal mutation for the amelanistic trait in corn snakes [34]. It corresponds to a 5832 bp Copia LTR-retrotransposon. The *CLCN2* and *OCA2* retrotransposon inserts only differ by a single nucleotide substitution (Additional File 6). It is therefore possible that this transposable element is currently active in the corn snake genome and contributes to the evolution of new traits. Using *megablast* (> 5000 bp out of 5832 bp matching with > 95% sequence identity), we found 85 copies of this retrotransposon in the wild-type corn snake Hi-C assembly. We interrogated the genomes of other snake species and only found copies within the *Pantherophis* genus and in the closely-related Pacific gopher snake (*Pituophis catenifer*—13.8 MYA [49]) suggesting a recent appearance and amplification of the Copia LTR-retrotransposon in the *Pantherophis*/*Pituophis* clade ( [50]; Fig. 5).

### *CLCN2* gene-editing produces Stripe-like corn snakes

Our analyses point to (i) a regulatory mutation impacting *CLCN2* as responsible for the Motley phenotype, and (ii) a disruptive mutation affecting the function of the Stripe CLCN2 protein. Using our established CRISPR-Cas9 gene-editing protocol for corn snakes [36], we generated *CLCN2* knock-outs. After validation on a corn snake fibroblast cell line, we selected a gRNA situated near the insertion site of the retrotransposon element in the *stripe* allele (Fig. 4C). The gRNA and the Cas9 protein were injected in 41 pre-vitellogenic follicles of two wild-type females that were crossed with a different wild-type male each. The females together laid 27 eggs, 22 of which hatched to give six mutated animals (Additional File 1: Table S3). As in our previous gene-editing experiments in corn snakes [35, 36], both the maternal and the paternal alleles were mutated suggesting that the Cas9/gRNA complex is still active after the formation of the zygote.

**Fig. 6:**
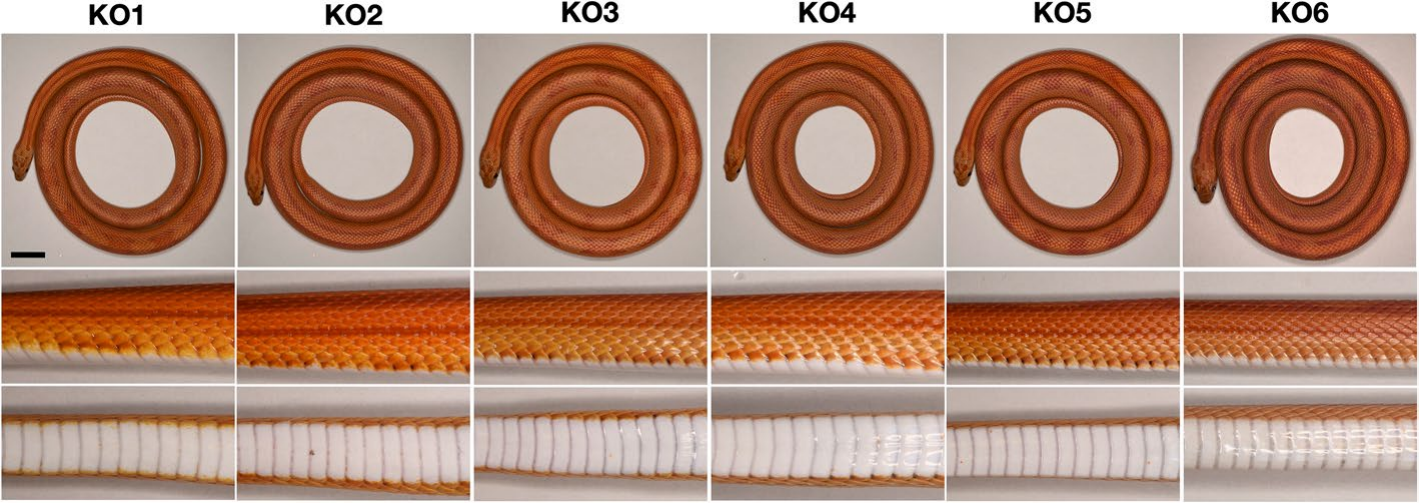
*CLCN2* gene-edited corn snakes. The dorsal, lateral, and ventral views of the six *CLCN2* knock-outs (KO) resemble that of Stripe corn snake. The ID/induced mutation correspondence can be found in Table S3 (Additional File 1). All animals were 1 year old at imaging. Scale bar: ~ 2 cm

The six mutated animals have a modified coloration pattern that strongly resembles the Stripe phenotype (Fig. 6; Additional File 1: Fig. S6). Although the dorsal pattern varies from one individual to the other, we primarily observe stripes at the anterior part of the body and fused dorsal blotches or blotches that take a butterfly-like shape at the posterior part. All *CLCN2* knock-outs lack the black ventral checkers, and their lateral blotches are replaced by lateral stripes that fade away posteriorly. Similarly to the Stripe individuals, the red pigmentation of the gene-edited animals increases with age and the blotches, if any, become less discernible (Additional File 1: Fig. S6). These results conclusively demonstrate that *CLCN2* is indeed involved in the skin color patterning process in corn snakes.

### *CLCN2* expression pattern in the embryonic skin

Our genomic, transcriptomic, and functional analyses support that *CLCN2* is a determinant of skin color pattern in corn snakes. The CLC family of Cl^−^ channels comprises nine members (*CLCN1-7*, *CLCNKA*, *CLCNB*) in Chordates, that can all be found in snakes as single-copy genes. Only *CLCNKA* is not annotated in the corn snake genome but is present in other snakes. It is likely that the genomic region harboring *CLCNKA* is absent in the CU assembly. In mammals, *CLCN2* is broadly-expressed, and codes for a plasma membrane channel involved in anion transport, which is activated by negative membrane voltage, cell swelling, or a rise in intracellular Cl^−^ concentration [51–54]. *CLCN2* deficiency in mice leads to vacuole formation in myelin sheets of central axons [54], as well as retina and male germ cell degeneration [52]. The migratory capacity of *CLCN2*-knock-down rat keratinocytes is reduced in vitro [55]. In humans, *CLCN2* mutations have been associated with leukoencephalopathy [56].

To our knowledge, *CLCN2* has been previously associated with skin coloration in a single study. Indeed, a differential gene expression study comparing the labyrinthine-patterned skin of the marble trout (*Salmo marmoratus*) and the spotted skin of the brown trout (*Salmo trutta*) identified *clcn2* as a strong candidate to explain the differences in their coloration pattern [57]. *Clcn2* is among the most significantly upregulated genes in the skin of the marble trout compared to the brown trout. No association of *CLCN2* with skin pattern defects has been observed in the zebrafish. The three copies of *clcn2* in the zebrafish have sub- and neofunctionalised; *clcn2a* is mostly expressed in the adult brain, eye, and heart, *clcn2b* is lowly expressed in most tissues, and *clcn2c* is expressed in a teleost-specific cell type, the ionocytes, and in the adult gills [58]. Mutations in the zebrafish *clcn2* genes have not been associated with variations in the skin coloration pattern.

**Fig. 7:**
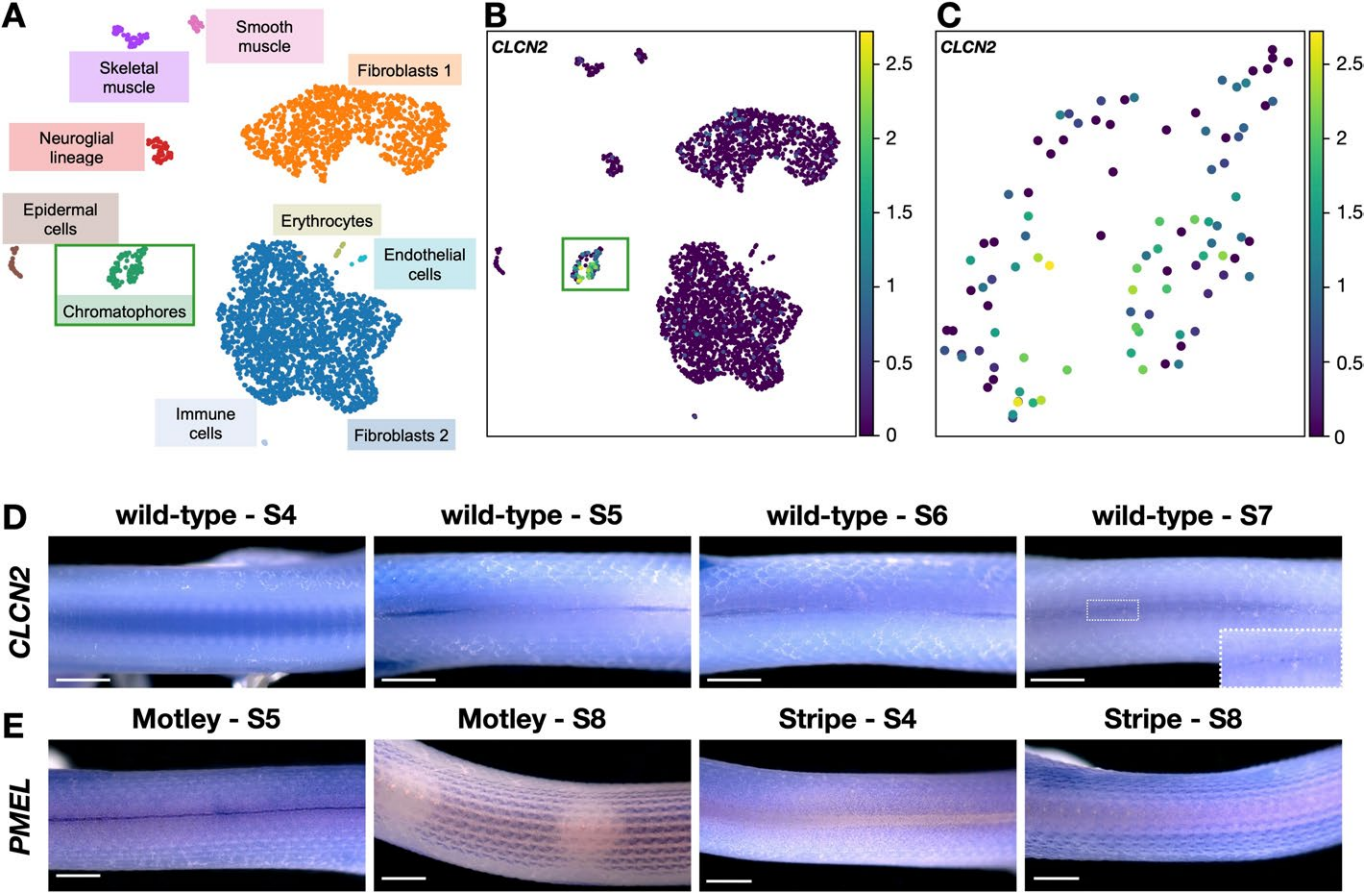
*CLCN2* expression in the embryonic skin.** A** Cell-type assignment of the nine clusters we identified in the embryonic skin of a wild-type corn snake based on single-cell transcriptomic analyses. Uniform manifold approximation and projection (UMAP) representation of (**B**) the overall expression level of *CLCN2* and (**C**) its expression within the chromatophores cluster. **D** Whole mount in situ hybridization of wild-type embryos with a species-specific *CLCN2* probe. Dorsal view of the anterior part of the embryos at developmental stages 4, 5, 6, and 7. *CLCN2* expression is barely detectable. Inset for S7: magnification of the region in the dashed rectangle. **E** WISH of Motley and Stripe embryos with a *PMEL* probe. At stages 4 and 5, we observe uniform expression. At stage 8, the pattern is already established with fused blotches and stripes visible on the Motley and Stripe embryos, respectively. Scale bars: 1 mm

We sought to find out if *CLCN2* is expressed by corn snake and zebrafish chromatophores. In a previous study, we produced single-cell data from the skin of a wild-type corn snake embryo at the developmental stage 9 (embryonic day 25), at which the patterning process is underway with chromatophore progenitors forming aggregates where the future dorsal blotches will be positioned [35]. Using this dataset, we find that the expression of *CLCN2* is restricted to the chromatophore progenitors cluster (Fig. 7A-C). To investigate the expression of *CLCN2* in the zebrafish, we interrogated the single-cell transcriptomic dataset previously produced by Saunders et al. [59] from post-embryonic neural crest-derived cells. To produce this dataset, the authors (i) fluorescently tagged the neural crest-derived cells expressing *Sox10* in zebrafish, (ii) dissected trunks or skins of staged, post-embryonic individuals (7.2–11.0 SSL (standardized standard length)), (iii) selected the *Sox10*-expressing cells using fluorescence-activated cell sorting, and (iv) submitted the selected cells to single-cell transcriptomic sequencing. From the three paralogs, only *clcn2b* was expressed by a few Schwann cells (Additional File 1: Fig. S7). *Clcn2a* was expressed by a single cell and *clcn2c* not at all. According to Wagner et al. [60] and the accompanying SPRING interactive tool [61], no expression could be detected for any of the *clcn2* paralogs in the zebrafish embryonic stages from 2 to 24 h post fertilization when embryonic chromatophores differentiate. In this study, the authors performed single-cell RNA sequencing on > 92,000 cells from zebrafish embryos collected during the first day of development.

To localize the *CLCN2* expression in the skin of corn snake embryos, we performed whole-mount in situ hybridizations (WISH) on wild-type embryos with a species-specific *CLCN2* probe, that spans exons 15 to 18 (Fig. 7D). No expression is observed at stage 4, whereas at the later stages (5 to 7), we only see faint staining along the dorsal midline forming a dashed line. The level of expression is probably at the detection limit of the hybridization protocol and thus not conclusive as to the localization of the *CLCN2* expression in the skin. Indeed, we measured low levels of expression both with our bulk RNA-seq (Fig. 3C) and single-cell transcriptomic sequencing (Fig. 7C). As a comparison, we provide WISH results with a species-specific *PMEL* (premelanosome protein) probe (Fig. 7E). In our previous studies on corn snakes and leopard geckos, we established that *PMEL* is expressed by all chromatophore progenitors at early stages of development [35, 62]. Similarly to the wild-type [35], at an early developmental stage (S4 and S5), a homogeneously-distributed *PMEL* staining is evident from each side of the dorsal midline both for Motley and Stripe embryos. At a later stage (S8), it is possible to discern the fused dorsal blotches on the Motley embryo, whereas stripes of *PMEL* expression are evident on the Stripe embryo.

### *CLCN2* expression pattern in the brain and the eyes

**Fig. 8:**
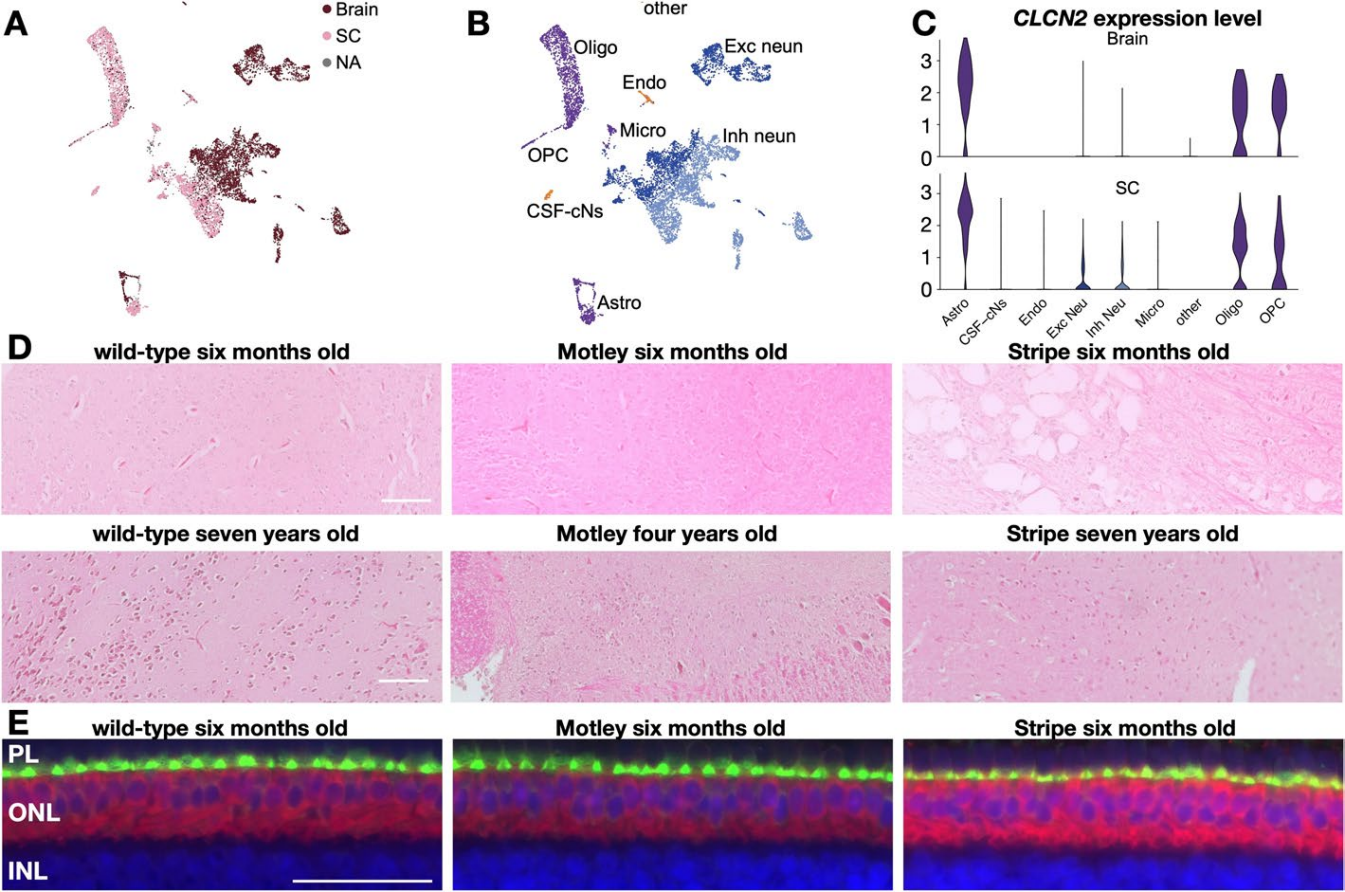
Single-nucleus transcriptomic analyses of a Stripe brain and histological analyses of Stripe and Motley brains and eyes.** A** UMAP representation of single-nucleus RNA-seq data from the brain and spinal cord of an adult Stripe animal color-coded by microdissected area after CMOs demultiplexing. “NA” cells could not be assigned to either tissue. **B** UMAP representation with a color-code to distinguish glial cells (purple), excitatory neurons (dark blue), inhibitory neurons (light-blue), and other cell types (orange). **C** Violin plots showing the expression of *CLCN2* in the different cell types in the brain and the spinal cord. **D** Hematoxylin/eosin-stained sections of the brain of juvenile and adult wild-type, Motley and Stripe animals. Vacuolation is visible in the brain of the juvenile Stripe individual. Scale bar: 100 μm. **E** Immunostaining of CLCN2 (red) and Ezrin (green) on eye sections from 6-month-old wild-type, Motley, and Stripe animals. CLCN2 labeled cells are situated in the outer nuclear layer (ONL) of the retina, but not the inner nuclear layer (INL). Ezrin labels the microvilli of Müller cells in the photoreceptors layer (PL). Nuclei are stained in blue with DAPI. Scale bar: 50 μm. CMO, cell multiplexing oligo; SC, spinal cord; Endo, endothelial cells; CSF-cN, cerebrospinal fluid contacting neurons; Oligo, oligodendrocytes; OPC, oligodendrocyte precursors; Micro, microglia; Astro, astrocytes; Exc neun, excitatory neurons; Inh neun, inhibitory neurons

As previously indicated, *CLCN2* disruptions result in brain dysfunctions in humans and mice. Motley and Stripe corn snakes do not show any signs of brain dysfunction, so we sought to find out if *CLCN2* is expressed in their brain and if they are histologically impacted by the corresponding mutations. We performed single-nucleus transcriptomic analyses on the anterior brain and the spinal cord of an adult Stripe corn snake (Fig. 8A, B, Additional File 1: Fig. S8), and we observed that *CLCN2* is primarily expressed in glial populations across the central nervous system as expected (Fig. 8C). In the mouse nervous system, HEPACAM (Hepatic And Glial Cell Adhesion Molecule, also known as GlialCAM) and MLC1 (Megalencephalic Leukoencephalopathy With Subcortical Cysts 1) stabilize CLCN2 in the plasma membrane by reducing its turnover rate and improving CLCN2 gating. Furthermore, they ensure its localization in cell–cell junctions. It has been shown that the presence of functional HEPACAM can even improve the function of mutated CLCN2 protein [56]. In corn snakes, we observe the co-expression of *HEPACAM*, *MLC1*, and *CLCN2* primarily in the Stripe astrocytes, but *HEPACAM* and *MLC1* are not expressed by wild-type chromatophores in the embryonic skin (Additional File 1: Fig. S9A, B). This implies that CLCN2 possibly functions in a different way in the chromatophores compared to the central nervous system. As we lack single-cell transcriptomic data from a wild-type corn snake brain, we analyzed existing bulk RNA-seq from adult male and female brain and cerebellum (SRA accessions: SRX6362408, SRX6362413, SRX6362424, SRX6362425) and confirmed the expression of *CLCN2* (8.83–26.43 transcripts per million (TPM)), *HEPACAM* (57.39–142.76 TPM), and *MLC1* (31.82–74.87 TPM) in these tissues as well (Additional File 1: Fig. S9C).

Histological comparison of wild-type, Motley, and Stripe brains of juveniles (6 months old) and adults (> 4 years old) reveals signs of vacuolation in the Stripe juvenile brain only (Fig. 8D). This observation corroborates our quantitative PCR results; as the expression of *CLCN2* is not significantly downregulated in Motley brain, a modified phenotype is not expected. The disruptive mutation in the *stripe* allele might be responsible for the vacuolation observed in the juvenile Stripe individual analyzed—but not the adult—although we have not observed any leukoencephalopathy symptoms in these individuals. Indeed, most of the symptoms observed in humans, such as loss of coordination, difficulty walking, personality changes, trouble speaking, and weak muscles, do not apply to snakes. Also, Stripe animals can reach advanced age; we currently keep a 15-year-old Stripe with normal behavior.

It has been shown that the male germ cells and the photoreceptors degenerate upon CLCN2 disruption in mice [52]. We thus performed immunostaining with a CLCN2 antibody on eye sections of wild-type, Motley, and Stripe 6-month-old animals. The photoreceptors and the outer nuclear layer did not show any signs of degeneration and CLCN2 protein expression was similar in the outer nuclear layer of all samples (Fig. 8E). Note that we have not observed any obvious sign of vision impairment in these animals. Furthermore, the respective mutations do not seem to affect the male germ cells as both Motley and Stripe corn snakes are fertile.

## Discussion

Analyses of zebrafish mutants [11] have uncovered some of the processes, such as cell-cell contacts, cell migration, and cell-substrate interactions, that control skin color pattern formation. Although these interactions can be efficiently modeled by the reaction–diffusion framework [10, 63, 64], much additional data will be required to understand the full complexity of these mechanisms, their potential universality, their developmental robustness, and the constraints associated to their evolvability. By characterizing the Motley and Stripe corn snake color morphs, we found that a chloride channel is involved in the patterning process of this species. Indeed, downregulated expression in Motley and disruption of the coding sequence of *CLCN2* in Stripe result in the transition from distinct blotches to elongated and merged blotches and to stripes, respectively. Thus, two different types of mutations on the same gene produce two different patterning phenotypes.

Using mapping-by-sequencing we identify a 4 Mb genomic interval where the Motley mutation resides and propose *CLCN2* as the main candidate gene within the interval. Our bulk transcriptomic analyses on embryonic skin, brain, and a body part suggest that, in Motley, different regulatory elements control the expression of *CLCN2* in different tissues and result in its downregulation specifically in the skin. In Stripe corn snakes, an LTR-retrotransposon element has been inserted in the *CLCN2* gene, thereby disrupting the coding sequence. We conclusively validate this finding with the generation of *CLCN2* knock-out corn snakes that have a phenotype similar to Stripe snakes. Based on our skin single-cell and brain single-nucleus transcriptomic results, we show that *CLCN2* is expressed in chromatophores, oligodendrocytes, and astrocyte cells. However, we hypothesize that CLCN2 interacts with HEPACAM and MLC1 in the brain but not in the skin. Investigation of publicly-available single-cell transcriptomic data from zebrafish chromatophores revealed the lack of *CLCN2* expression by these cells, although this gene was found differentially expressed in trouts with different skin coloration patterns. CLCN2 is a plasma membrane anion channel protein and its role in skin coloration is unknown. Chromatophores are considered as non-excitable cells, yet subtle changes in their membrane potential could influence their interactions during the patterning process [7]. We assume that CLCN2 plays an active role in this type of cell-cell communication, but further analyses are necessary to understand its exact function.

Terrazzo is another corn snake color morph that is characterized by dorsal and lateral stripes, similarly to the Stripe phenotype. We recently identified a disruptive mutation in *PMEL* responsible for the Terrazzo phenotype [35]. In both morphs, the patterning process seems to be arrested during development and the chromatophore progenitors fail to first form aggregates and then blotches, as observed in wild-type embryos. The main difference between Terrazzo and Stripe is the speckled and patternless coloration at the posterior part of the body in Terrazzo animals. This could be explained by the overall reduced number of progenitors in Terrazzo embryos [35], which does not seem to be the case in Stripe. Our current pattern characterization focuses on the dorsolateral coloration, but additional analyses are needed to understand the absence of black checkers on the ventral side of Stripe, Motley, and Terrazzo individuals. We assume that the ventral pattern depends on the proper formation of the dorsolateral one.

We observe that the same Copia LTR-retrotransposon is inserted in the *OCA2* gene of amelanistic [34] and the *CLCN2* gene of Stripe corn snakes, with only a single nucleotide difference. The appearance of the Copia LTR-retrotransposon could be a key driver in trait diversification in the *Pantherophis*/*Pituophis* clade given the great number of copies their genomes carry. Note that the insertion of retrotransposon elements has previously been reported to also impact human pigmentation [65] and coloration patterning in dogs [66].

We hypothesize that mutations similar to those uncovered in Motley and Stripe corn snakes could be determinants of pattern evolution in Squamates. For example, striped and blotched patterns can be found in pairs of closely-related species in other snake lineages, or even among populations of the same species, e.g., in the colubrids *Elaphe dione*, *Elaphe bimaculata*, *Lampropeltis getulus californiae*,* Zamenis situla*,* Thamnophis hammondii*, as well as in some boas and pythons, such as *Python regius* and *Boa constrictor*. Remarkably, in some species (e.g., *Elaphe quatuorlineata*), the color pattern changes from blotched to striped during post-embryonic development. The molecular mechanisms involved in this transition are unknown. We have recently shown in leopard geckos that the reduction in the number of iridophores is implicated in the transition from the juvenile bands to the adult spots [62]. We suggest that a similar phenomenon—such as a change in the survival or interaction properties of specific chromatophores—might be taking place in *Elaphe quatuorlineata*. The presence of different color patterns between pairs of closely related species could be associated with recent or ongoing speciation events and/or distinct behaviors. For example, some alleles affecting color patterns might be genetically coupled to anti-predator strategies. Indeed in the polymorphic Northwest garter snake (*Thamnophis ordinoides*), striped animals have a stronger propensity to flee, while unstriped and spotted conspecifics exhibit a more cryptic behavior [67]. Such a correlation could be due to genetic linkage between loci affecting behavior and pigmentation patterns. In the latter case, genetic linkage may be maintained by the presence of an inversion as is the case for alternative male morphs of the ruff shorebird showing differences in body size, behavior, and plumage pattern compared to the wild-type morph [68].

## Conclusions

Skin pigmentation is an excellent model to study pattern formation in biological systems. Studies in zebrafish have demonstrated the role of cell-cell communication mediated by potassium channels and gap junctions in regulating color pattern formation. Based on our genomic, bulk, and single-cell transcriptomic, functional, and developmental characterization of the Motley and Stripe corn snake color morphs, and their comparison with wild-type corn snakes, we suggest that skin color patterning in reptiles involves an anion channel, the chloride voltage-gated channel 2. Together with the extensive knowledge on zebrafish coloration, our systematic characterization of corn snake color morphs will allow us to build a consensus on the prevailing mechanisms for color pattern formation and identify novel components.

## Methods

### Animals and ethics statement

Corn snakes were housed and bred at the LANE animal facility running under veterinary cantonal permit no. 1008. Sampling, imaging, and gene-editing were performed under the experimentation permits GE/82/14, GE/73/16, GE24/33145, and GE150/34215 approved by the Geneva Canton ethical regulation authority.

### Genomic DNA next-generation sequencing

Genomic DNA was extracted from the parents and the offspring using the QIAGEN DNeasy Blood and Tissue kit (69504) following the manufacturer’s instructions. The TruSeq DNA PCR Free libraries were sequenced using an Illumina HiSeqX instrument producing 150 bp paired-end reads for family 1 and an Illumina HiSeq2000 producing 100 bp paired-end reads for family 2. We obtained 217 to 975 millions of paired-end reads per library. We checked the data quality and the presence of adapters with FASTQC [69]. We performed quality filtering with sickle v1.33 [70] and retained between 211 and 857 million reads, which corresponds to a 12.4 × –76.2 × average coverage for a 1.7 Gb genome.

### SNP calling and genomic interval mapping

The four genomic libraries (two for the parents and two for the offspring) of each family were aligned to the Hi-C and CU assembly of the corn snake genome [44] using bwa v0.7.17 [71] with default parameters in *mem* mode. The DNA Zoo team aligned the Illumina reads that we previously generated (UNIGE assembly; [37]) to the Hi-C scaffolds that originated from gDNA of a different individual, thus the final assembly is a hybrid. The CU assembly used gDNA from the same individual as the UNIGE assembly (Fig. 2B). We annotated the Hi-C assembly using GeMoMa v1.9 [72] and the NCBI annotation of the CU assembly (RefSeq GCF_029531705.1) as input. The original annotation for *CLCN2* was incomplete due to the presence of Ns in the CU assembly, so we used our RNA-Seq data to identify genomic regions corresponding to missing exons of the gene which were incorporated in the GeMoMa-created annotation file. We used SAMtools v1.9 [73] to (i) convert the output SAM files into BAM, (ii) remove duplicates using the *fixmate* mode with the *-m* flag and the *markdup* mode with the *-r* flag, and (iii) sort out the reads by their leftmost coordinates. Repetitive regions of the genome were identified using RepeatMasker v4.1.5 [74] and variants from these regions were ignored. We identified genomic variants with Platypus v0.8.1 [75] and retrieved the genomic interval where the Motley locus is located as previously described [37]. To account for the reference genome being heterozygous for Motley, variants were kept if they met the following criteria: 0/0 mapping for the homozygous samples and 1/0 or 0/1 for the heterozygous samples. The mapping was performed for each family separately and then for the two combined, comparing the cosegregating variants with the Motley locus in the four parental libraries to those from all libraries (parents and offspring). We predicted the impact of each co-segregating genomic variant found within the interval on genes and proteins using the SnpEff toolbox [76]. We further reduced the genomic interval by genotyping SNPs by Sanger sequencing and the primers given in Supplementary Table 4. The *CLCN2* transcript and genomic location were Sanger sequenced using the primers in Supplementary Table 4.

### Bulk RNA-seq sampling and analysis

We first extracted genomic DNA from a piece of the tail from each embryo and we genotyped them using the primers in Supplementary Table 4. We then extracted total RNA from the dorsal skin of four homozygous Motley and three wild-type embryos from one clutch and four homozygous Motley and two Stripe from another clutch using the Direct-zol RNA MiniPrep (Zymo Research, R2050). All samples had an RNA integrity number (RIN) ≥ 9.4. We sequenced between 37.9 and 40.1 million 100 bp paired-end reads from each TruSeq Stranded mRNA Library.

The bulk RNA-seq samples were aligned to the CU assembly (GCF_029531705.1.1) with STAR (v2.7.10b) [77] using default parameters for paired-end libraries. Gene expression quantification was performed using the featureCounts function implemented in the Subread package (v2.0.6) [78] counting uniquely mapped paired-end reads. Data normalization, transformation (considering variance stabilizing transformation), principal component analysis, and differential expression analyses with the Wald test and an FDR of 0.05 were performed with the DESeq2 package (v1.42.0) [79].

mRNA reads from Stripe individuals were mapped on the sequence of *CLCN2* with the insertion added between positions 605 and 1002 using bwa v0.7.17 [62]. Aligned reads were filtered to retain only those mapping on both the insertion and *CLCN2* by using their CIGAR string and alignment start position from the SAM files. We retained reads whose alignment started between − 80 and − 10 bp before the start or after the end of the insertion and the soft-mapped region was < 5 bp on both read ends.

### Real-time quantitative PCR

We performed the real-time quantitative PCR as previously described [35]. In short, we used 500 ng of RNA for the reverse transcription of each sample, all samples were run in triplicates and the primers are provided in Supplementary Table 4. We verified the PCR efficiency [80] for the amplification of *CLCN2* and *ALAS1*, the reference gene, and performed the statistical analyses as previously described [81].

### Gene-editing with CRISPR-Cas9

The anesthesia and the surgical procedure to access the ovaries and inject the follicles were performed as previously described [35]. Genomic DNA was extracted from the skin sheds of the offspring with the DNeasy Blood and Tissue Kit (69504, QIAGEN). We used the FastStart polymerase (12032902001, Sigma) to amplify the target regions. The gRNAs and the primers for the amplification of the target regions are detailed in Supplementary Table 4.

### Search of the Copia LTR retrotransposon

We performed a *megablast* search [82] with a threshold of minimum 5000 bp out of 5832 bp matching and 95% identity to detect the sequence of the Copia LTR retrotransposon element in the Hi-C corn snake assembly and the genomes of *Pantherophis spiloides* (GCA_037575465.1), *Pantherophis obsoletus* (GCA_012654085.1), *Pantherophis vulpinus* (GCA_037215915.1), *Pituophis catenifer* (GCA_029215685.1), *Arizona elegans* (GCA_022577455.1), *Ptyas mucosa* (GCA_012654045.1), *Coluber constrictor* (GCA_038048745.1), *Ahaetulla prasina* (GCA_028640845.1), *Thamnophis elegans* (GCA_009769535.1), *Notechis scutatus* (GCA_900518725.1), *Pseudonaja textilis* (GCA_900518735.1), *Crotalus tigris* (GCA_016545835.1), *Candoia aspera* (GCA_035149785.1), and *Python bivittatus* (GCA_000186305.2).

### Saunders et al. zebrafish single-cell transcriptomics

We downloaded the Saunders et al. single-cell data matrices from the NCBI GEO repository (GSE131136). We retained only euthyroid zebrafish samples from all time points available in the study. Using Scanpy v1.9.5, we filtered the data by discarding genes expressed in fewer than 3 cells and cells that exhibited expression of fewer than 300 genes, a mitochondrial UMI read percentage greater than 10% of total mapped reads, and UMI counts exceeding 15,000 to remove doublets. Additionally, we used Scrublet v0.2.3 to discard predicted doublets. The data was then normalized using sc.pp.normalize_per_cell with counts_per_cell_after set to 10,000, followed by normalization with sc.pp.log1p. We regressed out cell cycle genes using zebrafish orthologs of cell cycle genes obtained from the scverse GitHub (regev_lab_cell_cycle_genes.txt). PCA was computed with sc.tl.pca, and k-nearest-neighbor analysis was performed with sc.pp.neighbors using n_neighbors = 10 and n_pcs = 15. Batch correction was applied using sc.external.pp.bbknn. Finally, cell clusters and gene expression UMAP representations were generated using sc.tl.umap. Cell types were annotated based on the expression of marker genes (Additional file 1: Fig. S8) discussed in the original publication.

### Brain single-nucleus transcriptomics

A Stripe corn snake was frozen at − 20 °C after euthanasia. It was then thawed for the dissection of the brain and the spinal cord. The tissues were then flash-frozen. Nuclei were isolated using the Nuclei EZ Prep Kit (NUC-101, Sigma). Briefly, frozen tissue was resuspended in 2 mL of cold isolation buffer, homogenized with a KIMBLE Dounce tissue grinder (D8938, Sigma), and incubated at 4 °C during 5 min in the same buffer. The samples were centrifuged, resuspended, and incubated in fresh buffer for 5 extra minutes. Nuclei were washed twice using 1% BSA (bovine albumin serum) in PBS (phosphate-buffered saline) containing 50 U/ml of SUPERase-In (AM2696, Thermo Fisher) and 50 U/ml of RNasin (N2611Promega). Nuclei were resuspended in the appropriate volume of specific cell multiplexing oligos (CMO—CG000388, 10 × Genomics) and incubated for 5 min at room temperature. After adding 1.9 mL of 1% BSA-PBS, nuclei were centrifuged for 10 min at 4 °C and 500 × *g*. A second washing step was performed to remove the excess of CMOs. Nuclei were finally filtered through a 30-μm strainer and stained with Hoechst (Invitrogen H3570, 1:500) for 5 min. To remove debris and only retain good quality nuclei, we used fluorescence-activated nuclei sorting (FANS) on a Beckman Coulter MoFlo Astrios. CMO-labeled and sorted samples were pooled after sorting. We used approximatively 10,000 nuclei per sample and 42 μl of nuclei suspension was used for the 10 × Genomics snRNA-seq preparation according to the manufacturer’s instructions (10 × Genomics Chromium 3′ Gene Expression Kit 3.1). The cDNA libraries were quality-controlled using a 2100 Bioanalyzer and TapeStation from Agilent and sequenced using a HiSeq 2500 sequencer from the iGE3 platform at the University of Geneva. FASTQ files (gene expression and CellPlex) were used as inputs to the 10 × Genomics Cell Ranger multi pipeline (v3.0.2) with pre-mRNA transcript detection in the CU corn snake genome. The generated UMI count matrix was converted to a Seurat v4.0.1 object containing the CMO information and processed for quality controls. Only nuclei with more than 1500 detected genes and more than 2000 detected UMIs were retained. We ran the scDblFinder package (1.4.0) to exclude doublets. We then performed (i) gene count normalization to the total expression and log-transformation; (ii) highly variable genes detection, scaling, and principal component analysis; (iii) graph-based clustering (30 first PCs and a clustering resolution of 1.0); and (iv) UMAP calculation. Cell types were annotated based on the expression of marker genes [83] (Additional file 1: Fig. S9).

### Whole mount in situ hybridizations

We designed species-specific digoxigenin-labeled antisense riboprobes for *CLCN2* and *PMEL* (Supplementary Table 4). Embryos at different developmental stages were fixed in 4% paraformaldehyde and dehydrated in methanol. Whole mount in situ hybridizations (WISH) were performed as previously described [84]. Embryos were imaged using the VHX-6000 (Keyence).

### Histological analyses and immunohistochemistry

Brain samples were fixed in 4% PFA and dehydrated in ethanol before embedding in paraffin blocks. Seven-μm microtome sections of the brain were stained with hematoxylin and eosin. The eyes were fixed in 37.5% methanol/12.5% acetic acid and dehydrated in ethanol before embedding in paraffin blocks. Immunohistochemistry was performed on 7-μm paraffin sections using anti-CLCN2 (ab154798; Abcam) primary antibody and anti-Ezrin (ab4069; Abcam) with a 10-min antigen retrieval in 1 mM EDTA pH8.0–0.05% Tween-20 at 95 °C. Ezrin stains the microvilli of Muller cells in the retina [85]. We used Alexa Fluor 568- (A-11011 l; ThermoFisher Scientific) and Alexa Fluor 488-labelled (A-11001; Thermo Fisher Scientific) secondary antibodies, and nuclei were stained with ProLong Gold antifade reagent with DAPI (P36935; Invitrogen). Images were acquired with a Leica DM5500 fluorescence microscope.

## Supplementary Information

Additional file 1. Supplementary information. This file contains supplementary figures 1-9 and supplementary tables 1-4.

Additional file 2. Differential gene expression analyses between Motley and wild-type and between Motley and Stripe.

Additional file 3. Protein and transcript sequences of wild-type and Stripe *CLCN2*.

Additional file 4. Genomic region harbouring *CLCN2* in the corn snake genome.

Additional file 5. Annotation of *CLCN2* in the corn snake genome (modified CU assembly - sequence provided in Additional file 4).

Additional file 6. Alignment of the wild-type and Stripe intron where the LTR-retrotransposon insertion occurs. The sequence of the LTR-retrotransposon identified in Amelanistic corn snakes is provided for comparison.

## Data Availability

The datasets generated and analysed in the current study are available in NCBI as follows: genomic sequencing PRJNA1143197 [86], bulk RNA-seq GSE273807 [87], single-cell RNA-seq GSE262160 [88], single-nuclei RNA-seq GSE273631 [89].
