# Supplementary material for: Regulatory and disruptive variants in the CLCN2 gene are associated with modified skin color pattern phenotypes in the corn snake
Source: Genome Biol. 2025 Mar 26;26:73. doi: 10.1186/s13059-025-03539-0 (PMC11948899; doi:10.1186/s13059-025-03539-0)
Supplement: Supplementary file 1 — Additional file 1. Supplementary information. This file contains supplementary figures 1-9 and supplementary tables 1-4. [file 13059_2025_3539_MOESM1_ESM.pdf]

Supplementary Information for

Regulatory and disruptive variants in the *CLCN2* gene are associated with modified skin colour pattern phenotypes in the corn snake

Sophie A. Montandon[1,2¶], Pierre Beaudier[1¶], Asier Ullate-Agote[1,3¶], Pierre-Yves Helleboid[1], Maya Kummrow[4], Sergi Roig-Puiggros[5], Denis Jabaudon[5,6], Leif Andersson[7,8], Michel C. Milinkovitch[1*], Athanasia C. Tzika[1*]

[1]Laboratory of Artificial and Natural Evolution, Department of Genetics & Evolution, University of Geneva, Switzerland
[2] current address: Bracco Suisse S.A., Plan-les-Ouates, Switzerland
[3] current address: Biomedical Engineering Program, Center for Applied Medical Research (CIMA), Universidad de Navarra, Instituto de Investigación Sanitaria de Navarra (IdiSNA), Pamplona, Spain
[4] Tierspital, University of Zurich, Zurich, Switzerland
[5] Department of Basic Neurosciences, University of Geneva, Geneva, Switzerland
[6] Clinic of Neurology, Geneva University Hospital, Geneva, Switzerland
[7] Department of Medical Biochemistry and Microbiology, Uppsala University, Uppsala, Sweden
[8] Department of Veterinary Integrative Biosciences, Texas A&M University, College Station, Texas, USA

¶ These authors contributed equally to this work

*Corresponding authors:
E-mail: athanasia.tzika@unige.ch (ACT)
E-mail: michel.milinkovitch@unige.ch (MCM)

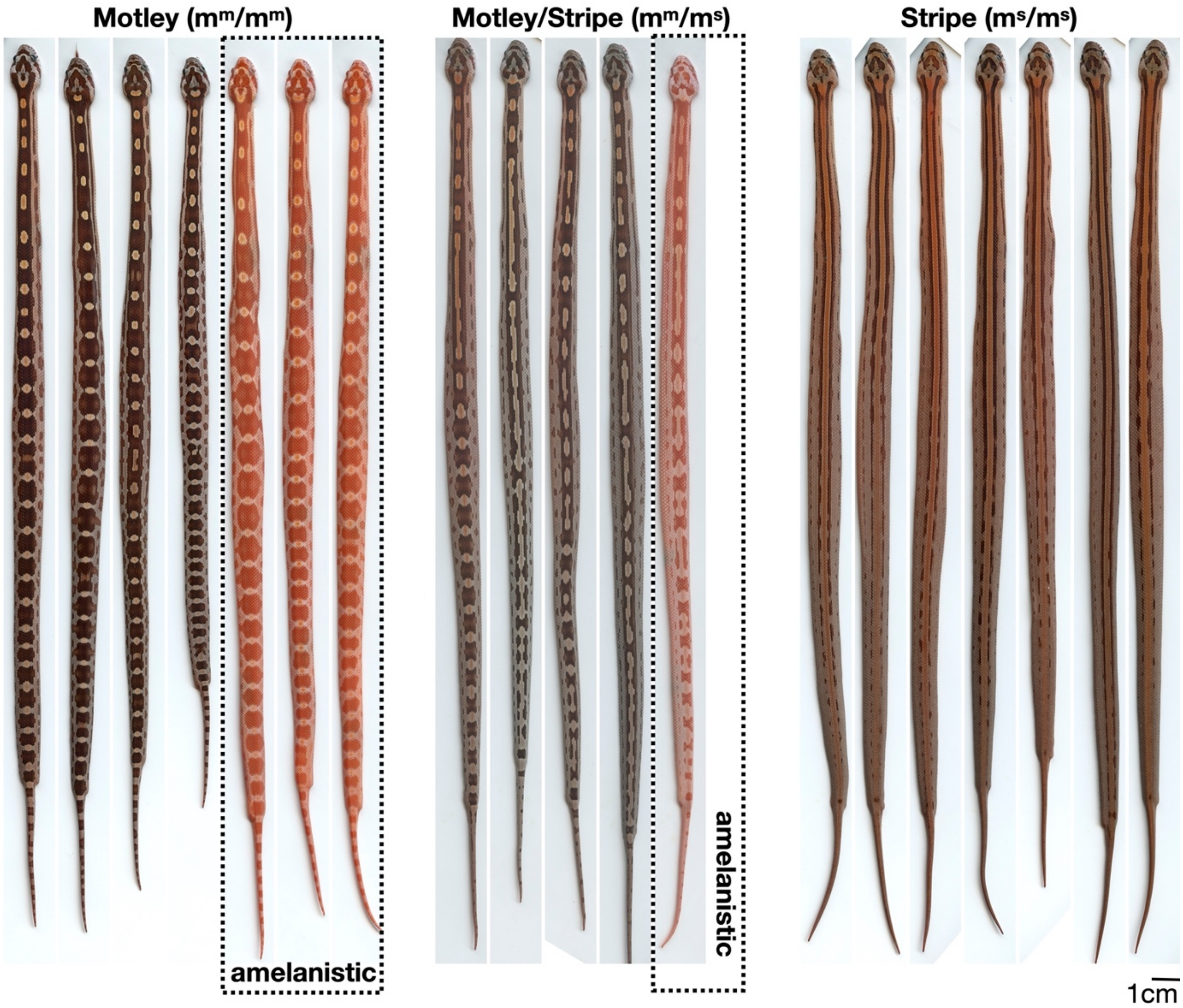

Fig. S1. Pattern variability of the Motley, Motley/Stripe, and Stripe phenotypes. Dorsal view of Motley, Motley/Stripe and Stripe hatchlings.

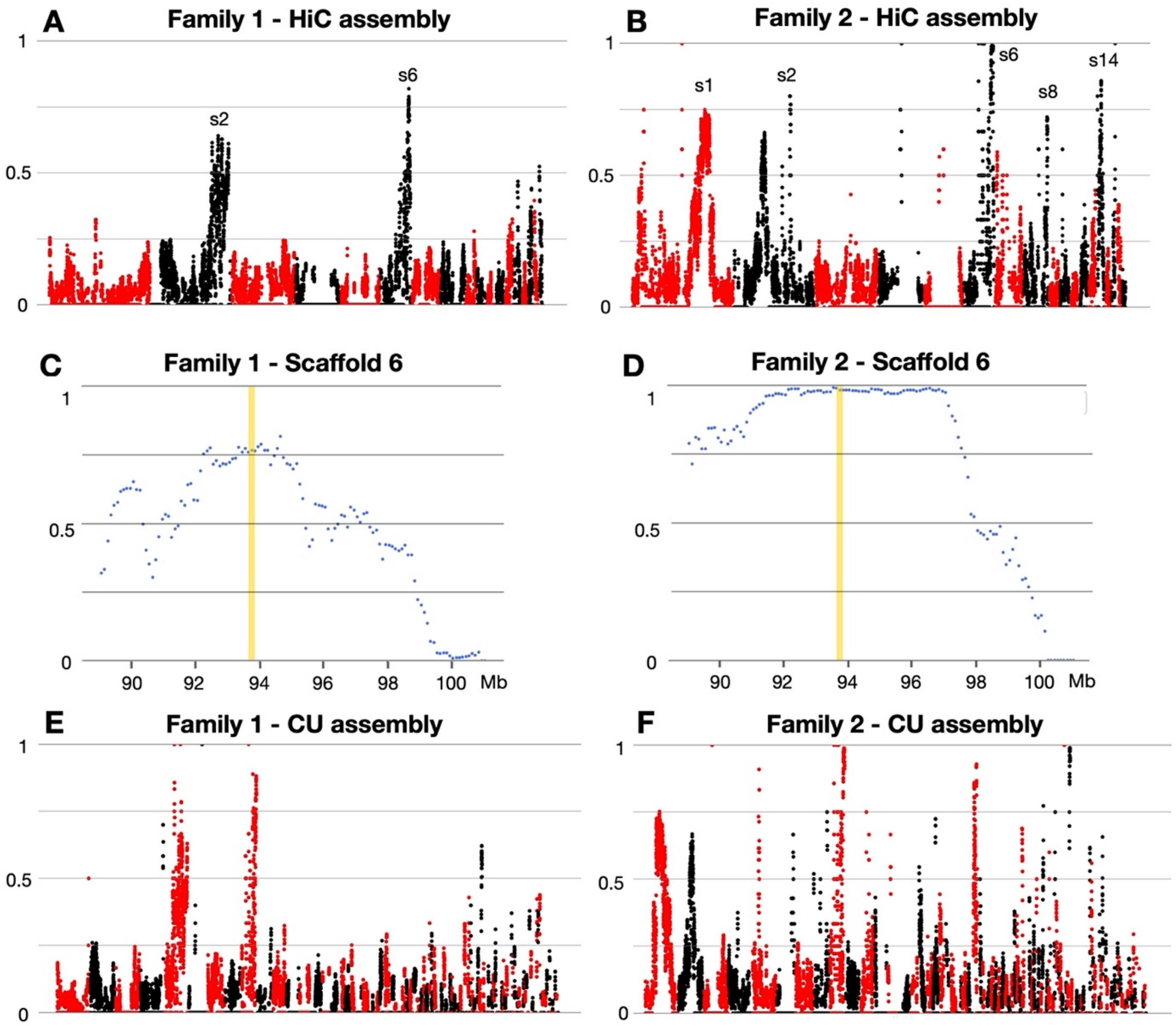

Fig. S2. Mapping of the Motley variant with family 1 and family 2 separately. Proportion of co-segregating variants with the Motley locus (y-axis) in family 1 (**A**) and family 2 (**B**) when mapped separately to the HiC assembly. Proportion of co-segregating variants in family 1 (**C**) and family 2 (**D**) within the interval on Scaffold 6 (60.1-64.1 Mb) of the Hi-C assembly. Proportions are calculated with a 1-Mb sliding window and a step of 100 Kb. The position of the main candidate gene (*CLCN2*) is highlighted in yellow. Proportion of co-segregating variants with the Motley locus (y-axis) in family 1 (**E**), family 2 (**F**) when mapped separately to the CU assembly. Proportions are calculated with a 1-Mb sliding window and a step of 100 Kb. Scaffolds are alternatively coloured in red and black.

A

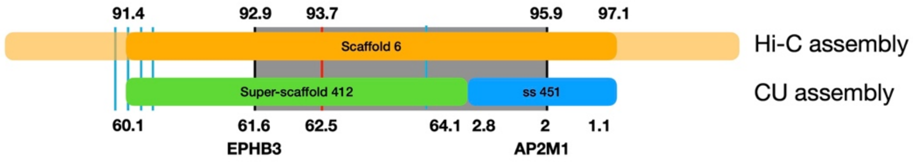

B

Individual	ATP11B	LAMP3	RCF4	SENP5	EPHB3	CHRD	PSMD2	AP2M1
EG.266.41.1	no	na	na	na	na	na	na	no
EG.266.41.2	no	na	na	na	na	na	na	no
EG.266.41.4	no	na	na	na	na	na	na	no
EG.266.41.6	no	na	na	na	na	na	na	no
EG.266.41.7	no	na	na	na	na	na	na	no
EG.266.41.8	no	na	na	na	na	na	na	no
EG.266.41.9	no	na	na	na	na	na	na	no
EG.266.41.10	no	na	na	na	na	na	na	no
EG.266.41.11	no	na	na	na	na	na	na	no
EG.266.41.12	no	na	na	na	na	na	na	no
**EG.266.41.13**	**yes**	**yes**	**yes**	**yes**	**yes**	no	na	no
EG.266.41.14	no	na	na	na	na	na	na	no
EG.266.41.17	no	na	na	na	na	na	na	no
EG.266.41.18	no	na	na	na	na	na	na	no
**EG.266.41.19**	no	na	na	na	na	na	no	**yes**
EG.266.41.20	no	na	na	na	na	na	na	no
EG.266.41.21	no	na	na	na	na	na	na	no
EG.266.41.22	no	na	na	na	na	na	na	no
EG.266.41.23	no	na	na	na	na	na	na	no
EG.266.41.24	no	na	na	na	na	na	na	no
**EG.266.41.25**	no	na	na	na	na	na	no	**yes**
**EG.266.41.26**	no	na	na	na	na	na	no	**yes**

Individual	ATP11B	PSMD2	AP2M1
EG.266.418.1	no	na	no
EG.266.418.3	no	na	no
EG.266.418.6	no	na	no
EG.266.418.8	no	na	no
**EG.266.418.9**	no	na	**yes**
EG.266.418.10	no	na	no
EG.266.418.12	no	na	no
EG.266.418.13	no	na	no
EG.266.418.14	no	na	no
EG.266.418.15	no	na	no

Fig. S3. Reduction of the genomic interval harbouring the Motley variant. A Schematic representation of Scaffold 6 of the Hi-C assembly and Super-scaffolds 412 and 451 of the CU assembly. The blue bars indicate the positions of the genotyped fragments, located within the coding regions of the genes shown in the table below. The two black bars indicate the position (in Mb) of the recombination events closest to *CLCN2*. These two bars mark the reduced interval, that we highlight in dark grey. The red bar marks the position of *CLCN2*. **B** List of the 32 genotyped individuals and the genotyping results. Recombined sites are highlighted in red in the table and 'na' corresponds to sites that were not genotyped.

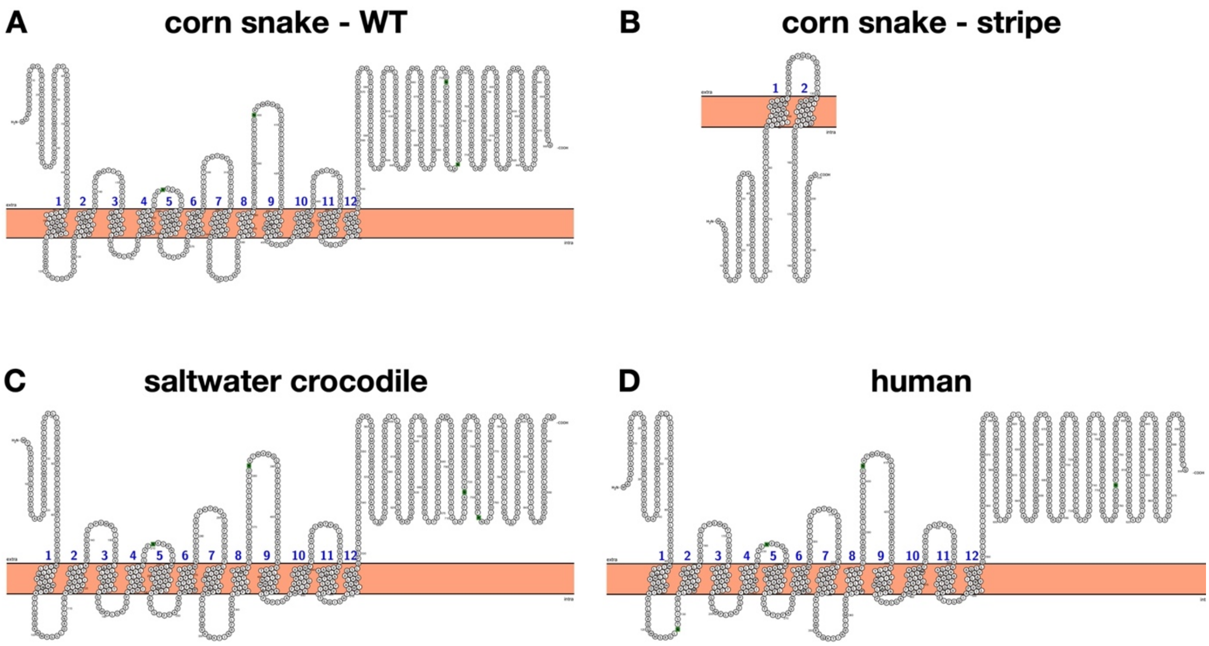

Fig. S4. Transmembrane domains of *CLCN2*. Schematic representation of transmembrane domains of *CLCN2* from (**A**) a WT corn snake, (**B**) a Stripe corn snake, (**C**) a saltwater crocodile (Ensembl transcript ENSCPRT00005018043.1), and (**D**) a human (Ensembl transcript ENST00000265593.9) generated with PROTTER.

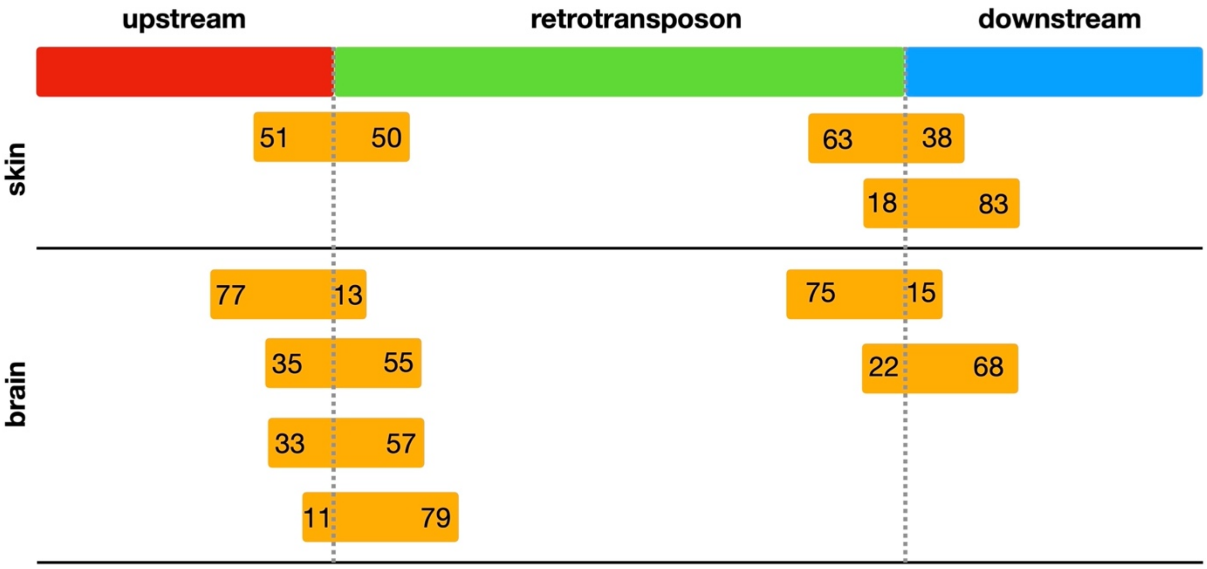

Fig. S5. Sequencing reads spanning the retrotransposon element and its flanking regions. We aligned Stripe RNA-seq reads from embryonic skin to the Stripe *CLCN2* transcript including the 397 bp insertion and identified three reads that span the retrotransposon element and its flanking regions. We also aligned the single-nuclei RNA-seq reads from a Stripe adult brain and obtained six such reads. Numbers correspond to the number of nucleotides that aligned before and after the dashed grey lines that mark the boundaries of the insertion.

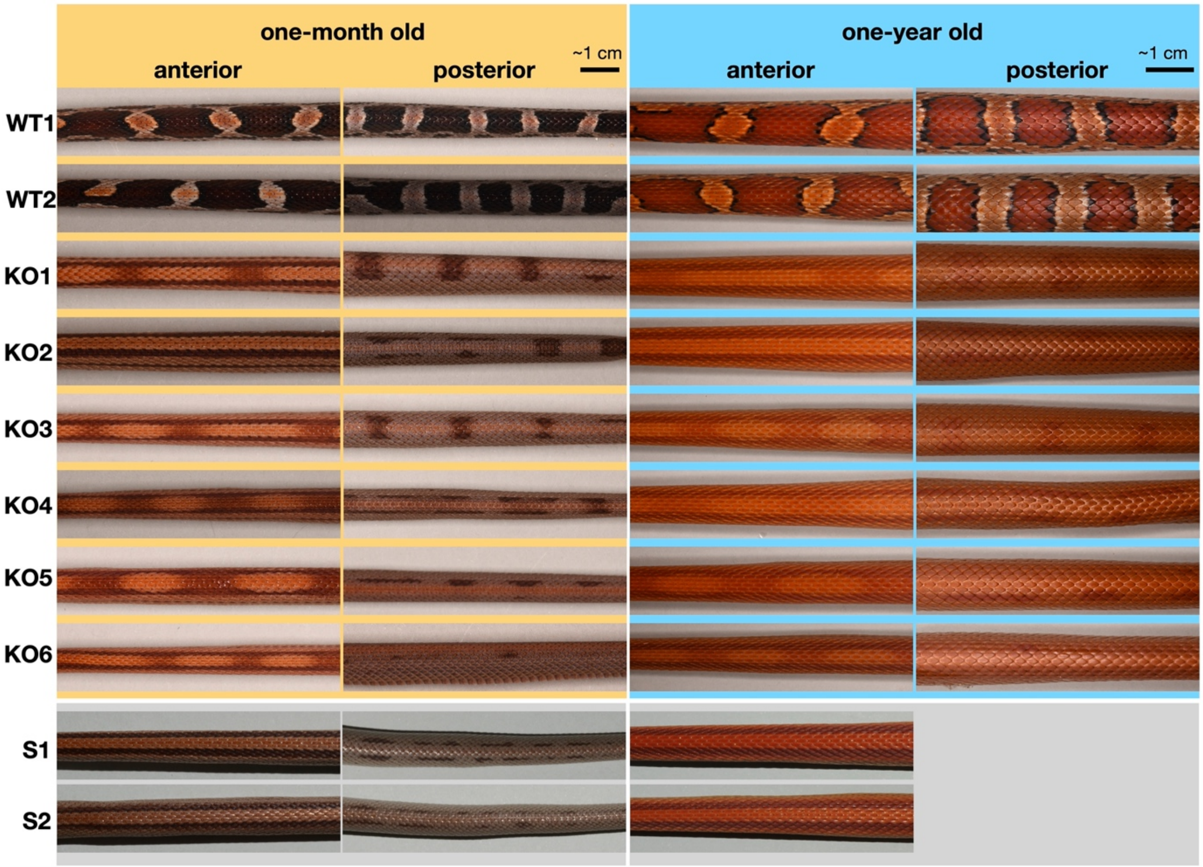

Fig. S6. Pattern variability of the six *CLCN2* knock-outs. Dorsal photos of the anterior (near the head) and the posterior (near the tail) body of the knock-out (KO) animals at one-month old and at one-year old. For comparison, we provide images of wild-type (WT-top) and Stripe (S-bottom) animals.

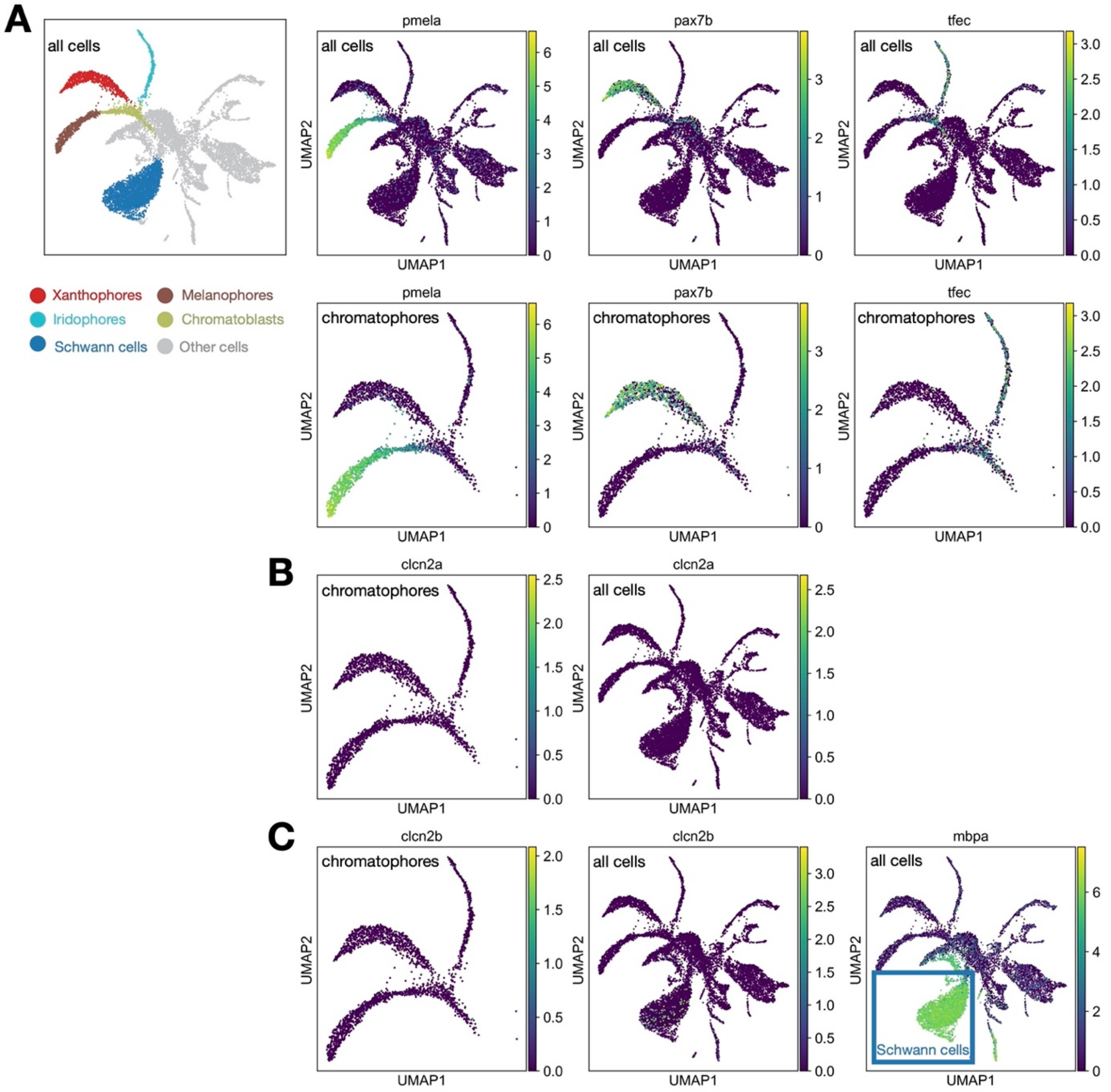

Fig. S7. *CLCN2* expression in zebrafish post-embryonic neural-crest derived cells. Reanalysis of the data from Saunders et al. 2019. **A** Cell-type assignment of the chromatophore-related clusters based on the expression of marker genes discussed in the original publication (*pmela* for melanophores, *pax7b* for xanthophores, and *tfec* for iridophores). **B** Lack of *clcn2a* expression in the chromatophores and all the other cell types. **C** Lack of *clcn2b* expression in chromatophores and detectable expression in Schwann cells. This cell type is identified by the expression of *mbpa*, as shown in the original publication.

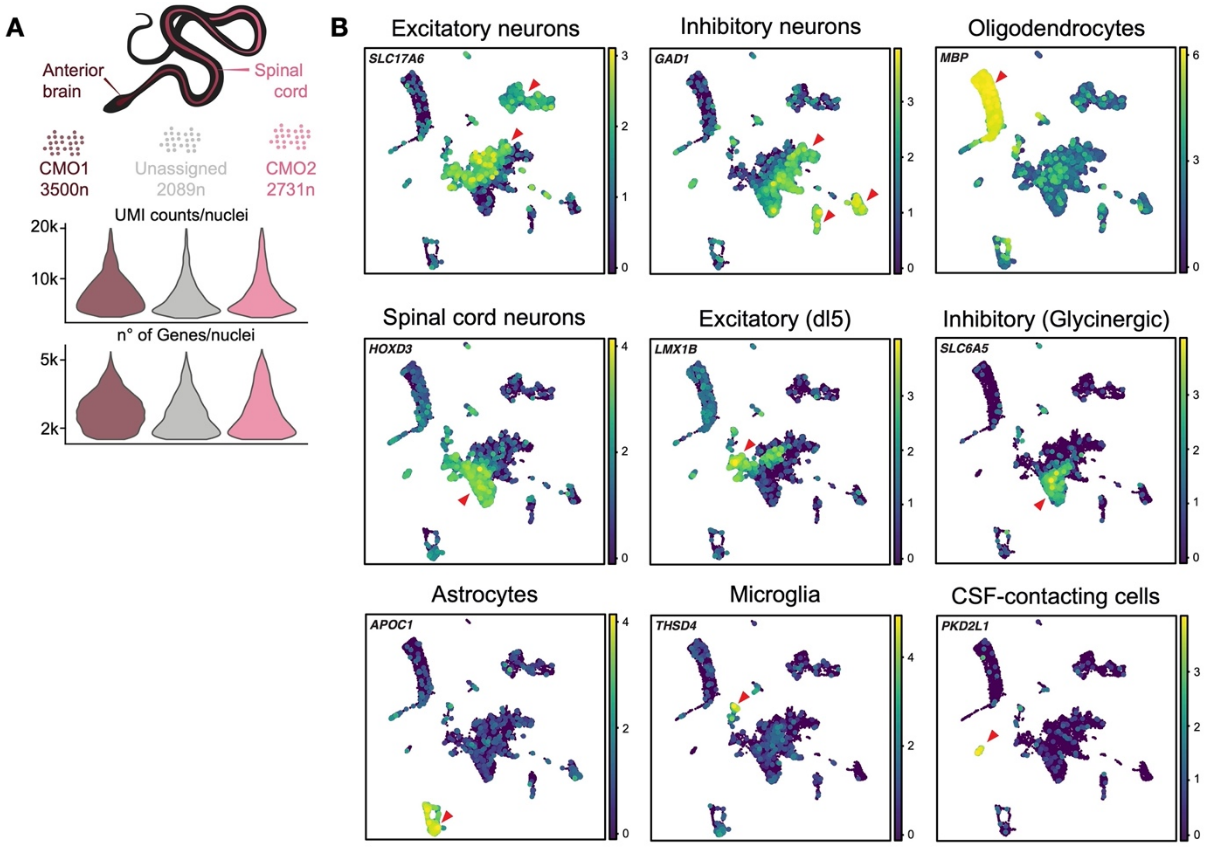

Fig. S8. Diversity and molecular identities of adult central nervous system cells from a Stripe individual. A *Top:* Number of collected nuclei after capture and quality control processing. *Bottom:* UMI counts and genes per nucleus plots in the demultiplexed samples after quality control. **B** Expression plots of specific markers used to annotate the cell types. Red arrows point to the cells primarily expressing each marker gene. n: nuclei, CMO: cell multiplexing oligo, CSF: cerebrospinal fluid.

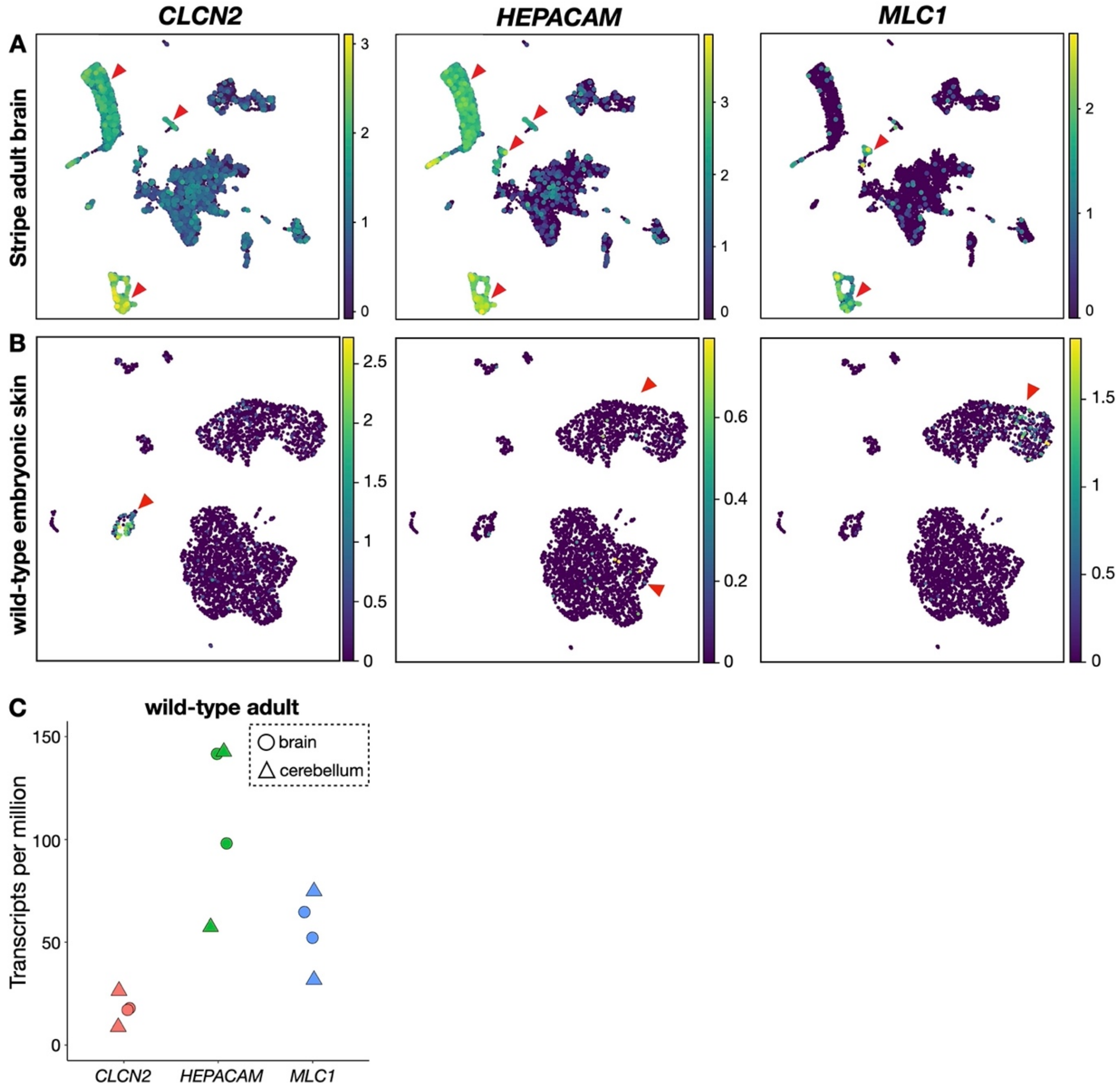

Fig. S9. Expression of proteins interacting with CLCN2. Comparison of the expression of *CLCN2*, *HEPACAM*, and *MLC1* in the central nervous system of a Stripe adult (**A**) and in the wild-type embryonic skin (**B**). Red arrows point to the cells primarily expressing these genes. (**C**) Expression level of *CLCN2*, *HEPACAM*, and *MLC1* in adult male and female brain and cerebellum from wild-type animals. The transcripts per million were calculated from bulk RNAseq data.

Supplementary Table 1. Genomic DNA libraries used for mapping the Motley causative variant. In parentheses, we provide the total number of individuals in each library or their ID, if there is only one (column 'Source'), and the average coverage for a 1.7 Gb genome (column 'Filtered reads'). PE: Paired-end reads.

Source	Family	Genotype	Library type	Total reads	Filtered reads
Motley female (EG41)	1	m^m/m^m	150 bp Illumina PE	487,581,366	428,960,400 (75.7x)
WT male (EG266)	1	m^m/+	150 bp Illumina PE	425,172,973	383,108,359 (67.6x)
Motley offspring (14)	1	m^m/m^m	150 bp Illumina PE	296,053,038	290,818,704 (51.3x)
WT offspring (10)	1	m^m/+	150 bp Illumina PE	221,345,019	217,374,619 (38.4x)
Motley female (EG54)	2	m^m/m^m	100 bp Illumina PE	129,955,259	127,040,500 (14.9x)
WT male (EG266)	2	m^m/+	100 bp Illumina PE	108,610,285	105,609,252 (12.4x)
Motley offspring (18)	2	m^m/m^m	100 bp Illumina PE	185,627,176	184,019,891 (21.2x)
WT offspring (18)	2	m^m/+	100 bp Illumina PE	173,748,776	172,504,929 (20.3x)

Supplementary Table 2. List of the 77 protein coding genes in the Motley interval on the CU assembly (scaffolds 412 and 451). We provide information on the presence of co-segregating mutations in the coding region of each gene, the number of amino-acid modifications in the protein coding part of the genes ('aa mod' column), the presence of high impact mutations, such as the introduction of STOP codons, deletions/insertions, and mutations on intron donor and acceptor sites ('high impact' column), and the number of variants within introns ('intron variants' column). We also indicate the log2 fold-change and the *p*-value from the differential expression analyses (comparison of Motley vs. wild-type). We highlight in red *CLCN2*, the main candidate gene, in bold *EPHB3* and *AP2M1*, which are situated at the boundary of the reduced interval, and in yellow the differentially expressed genes in the embryonic skin of motley embryos compared to wild-type. Genes with a 'NA' fold-change are not expressed in our RNA dataset, and genes with 'NA' *p*-value are of low abundance.

Start	End	Gene name	CDS variants	aa mod	High impact	Intron variants	Fold change	*p*-value
				**Scaffold 412**				
60154517	60384696	*MCF2L2*	Yes	/	/	14	0.718	0.999
60244044	60263437	*B3GNT5*	/	/	/	0	0.874	0.999
60395822	60418632	*RFC4*	/	/	/	0	-0.006	0.999
60421534	60431581	*EIF4A2*	/	/	/	0	-0.016	0.999
60447217	60482676	*KNG1*	/	/	/	1	NA	NA
60513538	60531534	*HRG*	Yes	0	/	0	NA	NA
60534679	60560586	*LOC117678772 (uncharacterized)*	/	/	/	0	NA	NA
60564673	60576750	*LOC117678138 (fetuin-B-like)*	/	/	/	0	NA	NA
60589385	60600568	*LOC117678790 (fetuin-B-like)*	/	/	/	0	NA	NA
60610570	60624650	*LOC117678771 (fetuin-B-like)*	/	/	/	0	NA	NA
60634233	60644692	*AHSG*	/	/	/	0	NA	NA
60683030	60731412	*SENP5*	/	/	/	28	0.158	0.999
**61607010**	**61750347**	***EPHB3***	**/**	**/**	**/**	**3**	**$6.37e^{-05}$**	**0.999**
62255511	62291127	*LOC117679779 (MFSD8-like)*	/	/	/	3	-0.290	0.999
62291132	62305720	*POLR2H*	/	/	/	6	-0.110	0.999
62310135	62322549	*EPO*	/	/	/	6	1.985	1
62355342	62423871	*CHRD*	Yes	0	/	16	0.295	0.999
**62470959**	**62619563**	***CLCN2***	**/**	**/**	**/**	**53**	**-2.314**	**$8.88e^{-11}$**
62624846	62633854	*LOC117679760 (HSBP7-like)*	Yes	0	/	2	NA	NA
62643816	62751401	*FAM131A*	/	/	/	4	NA	NA
62819776	62967511	*EIF4G1*	Yes	0	/	32	-0.143	0.999
62970772	63016030	*PSMD2*	/	/	/	7	-0.054	0.999
63039705	63128476	*ECE1*	/	/	/	3	-0.059	0.999
63136561	63152245	*LOC117679774 (MUL1-like)*	/	/	/	1	0.071	0.999
63240528	63244709	*CAMK2N2*	/	/	/	0	1.186	1
63268820	63316032	*EEF1AKMT4*	Yes	0	/	4	-0.645	0.051
63319570	63338890	*ALG3*	Yes	0	/	1	-0.498	0.999
63342262	63423600	*VWA5B2*	Yes	1	/	4	-0.459	0.999
63423746	63443647	*LOC117679770 (uncharacterized)*	/	/	/	0	0.250	0.999
63445444	63455978	*LOC117679782 (PHF-13-like)*	/	/	/	1	-0.060	0.999
63468471	63507332	*ABCF3*	Yes	0	/	18	-0.213	0.999
63519208	63565770	*LOC117679781 (CYP2J2-like)*	/	/	/	11	NA	NA

63589344	63604792	*LOC117679768 (CYP2J4-like)*	/	/	/	1	NA	NA
63630130	63658661	*LOC117679759 (CYP2C31-like)*	Yes	1	/	5	NA	NA
63664296	63677919	*LOC117679763 (CYP2J5-like)*	/	/	/	0	0.737	NA
63678998	63696522	*LOC117679767 (CYP2J2-like)*	/	/	/	0	NA	NA
63705668	63723947	*LOC117657165 (CYP2J2-like)*	/	/	/	6	NA	NA
63735666	63759868	*LOC117679764 (CYP2J2-like)*	/	/	/	0	NA	NA
63782019	63826209	*LOC117679762 (CYP2J5-like)*	/	/	/	0	NA	NA
63829359	63854158	*LOC132708857 (CYP2J5-like)*	/	/	/	1	-0.637	NA
63870390	63905828	*LOC117679765 (CYP2J5-like)*	Yes	2	/	11	NA	NA
63880311	63962781	*LOC132709391 (CYP2*AB1*-like)*	Yes	2	/	24	NA	NA
63922763	63960094	*LOC117659711 (CYP2J5-like)*	/	/	/	12	NA	NA
63999199	64018407	*LOC117660148 (CYP2H2-like)*	/	/	/	4	NA	NA
**Scaffold 451 (reversed)**								
2767424	2776702	*LOC132709909 (CYP2D14-like)*	/	/	/	1	NA	NA
2726767	2759601	*LOC117658724 (CYP2J2-like)*	/	/	/	11	NA	NA
2547737	2723036	*LOC117660245 (CYP2J5-like)*	/	/	/	11	NA	NA
2482303	2523686	*LOC117660251 (CYP2J5-like)*	/	/	/	2	NA	NA
2441388	2466151	*LOC117660243 (CYP2J2-like)*	/	/	/	0	NA	NA
2391513	2406584	*LOC132709908 (CYP2J2-like)*	/	/	/	0	NA	NA
2358905	2373520	*LOC117660239 (CYP2J5-like)*	/	/	/	0	-1.53	0.452
2347900	2349939	*LOC132709912 (uncharacterized)*	/	/	/	0	NA	NA
2317381	2340541	*LOC117660238 (CYP2J5-like)*	/	/	/	0	NA	NA
2271782	2303612	*LOC117660240 (CYP2J2-like)*	/	/	/	0	NA	NA
2241359	2266789	*LOC117660244 (CYP2J4-like)*	/	/	/	2	NA	NA
2181615	2220855	*LOC117660246 (CYP2J5-like)*	/	/	/	0	NA	NA
2148866	2177468	*LOC117660241 (CYP2J5-like)*	/	/	/	3	NA	NA
2138489	2141350	*LOC117659188 (PCDHGB5-like)*	/	/	/	0	-0.05	NA
2092638	2120685	*LOC117660242 (CYP2C20-like)*	/	/	/	0	NA	NA
**2031066**	**2069070**	***AP2M1***	**/**	**/**	**/**	**1**	**-0.014**	**0.999**
1970724	1974450	*LOC132709911 (uncharacterized)*	/	/	/	0	NA	NA
1944904	2025430	*DVL3*	/	/	/	6	-0.154	NA
1876236	1928401	*EIF2B5*				4	-0.019	NA
1817325	1820266	*LOC117656336 (uncharacterized)*	/	/	/	16	NA	NA
1793727	1869572	*ABCC5*	/	/	/	34	0.001	0.999
1754016	1789321	*RNPEPL1*	Yes	0	/	3	-0.14	0.999
1715649	1750926	*CAPN10*	Yes	0	/	0	0.25	0.999

1700733	1706212	*LOC117659655 (CYP2D28-like)*	/	/	/	0	NA	NA
1635192	1666718	*LOC117659652 (CYP2J6-like)*	/	/	/	1	NA	NA
1599967	1626960	*LOC117659653 (CYP2J2-like)*	/	/	/	0	-0.62	0.001
1551851	1567323	*LOC117659654 (CYP2J4-like)*	/	/	/	0	-0.60	NA
1513234	1546265	*LOC117659651 (CYP2J2-like)*	/	/	/	0	NA	NA
1434028	1482852	*EIF4E2*	/	/	/	3	0.108	0.999
1379281	1416828	*CHRNG*	Yes	0	/	6	-1.244	5.08E-14
1340355	1369905	*CHRND*	/	/	/	0	0.129	0.999
1158576	1300214	*LOC117658614 (SLC12A9-like)*	/	/	/	15	-0.073	0.999
1062264	1120759	*ECEL1*	Yes	0	/	0	0.143	0.999

Supplementary Table 3. ***CLCN2*** **gene-editing results.** We provide information on the sequence modifications and phenotype of all the offspring. Highlighted in bold are the heterozygous individuals.

ID	Sample	gRNA	Sequence modifications	Phenotype
KO1	EG.17.1112.2023.1	578r	c.613_617del - p.F205SfsX226	modified
**KO2**	**EG.17.1112.2023.2**	**578r**	**c.[607_648del + 54bp int 5]+ [616_617insT] – p.[?]+[H207SfsX228]**	**modified**
WT1	EG.17.1112.2023.3	578r	None	WT
	EG.17.1112.2023.4	578r	None	WT
	EG.17.1112.2023.5	578r	None	WT
	EG.17.1112.2023.6	578r	None	WT
	EG.17.1112.2023.7	578r	None	WT
	EG.17.1112.2023.8	578r	None	WT
	EG.17.1112.2023.9	578r	None	WT
	EG.17.1112.2023.10	578r	None	WT
	EG.17.1112.2023.11	578r	None	WT
	EG.17.1112.2023.12	578r	None	WT
KO3	EG.378.1097.2023.1	578r	c.613_617del - p.F205SfsX226	modified
KO4	EG.378.1097.2023.2	578r	c.616_617insT - p.H207SfsX228	modified
KO5	EG.378.1097.2023.3	578r	c.616_617insT - p.H207SfsX228	modified
**KO6**	**EG.378.1097.2023.4**	**578r**	**c.[616_621del + 622A>T]+[616_618del] – p.[V206_H207del + I208L]+[V206del]**	**modified**
WT2	EG.378.1097.2023.5	578r	None	WT
	EG.378.1097.2023.6	578r	None	WT
	EG.378.1097.2023.7	578r	None	WT
	EG.378.1097.2023.8	578r	None	WT
	EG.378.1097.2023.9	578r	None	WT
	EG.378.1097.2023.10	578r	None	WT

Supplementary Table 4. Primers and gRNAs used in this study.

Purpose	Orientation	Name	Sequence
CLCN2 transcript amplification	Forward	CLCN2_F8	AGTCATTGGACTGACCTGT
(insertion site)	Reverse	CLCN2_R10	ATGTGCGGTTATCAAACAAGG
LTR amplification (gDNA)	Forward	CLCN2_F8b	GTGAGGAGGGGTCTGGGGTCT
	Reverse	CLCN2_R11	ACTTCAACTCCCAGAATTCCT
Genotyping	Forward	EG_ATP11B_F	CCAAACTTTTCTCCCACCCC
	Reverse	EG_ATP11B_R	CTACCCGAGTCAACAGGTGT
	Forward	EG_LAMP3_F	CAGAGAGCACCACTTCCAGA
	Reverse	EG_LAMP3_R	TCCAGTTTGACTTGTTGGCG
	Forward	EG_RCF4_F	GCTAAAATGAAAGCGACAAGATG
	Reverse	EG_RCF4_R	TGGTTCTGATGAGTTCTTGCAG
	Forward	EG_SENP5_F	AATTTCACCACTGCCCTCTG
	Reverse	EG_SENP5_R	AGCGTAACTCTCCTTGGGAC
	Forward	EG_EPHB3_geno_F1	ATCCATTCCCCATCTCCGTT
	Reverse	EG_EPHB3_geno_R1	CACCCAGTTTATCCAGCGC
	Forward	EG_CHRD_F	CAGGTAGCCGGAACAGGAA
	Reverse	EG_CHRD_R	TATCTGTCCGCGCAATTCTC
	Forward	EG_PSMD2_F	GAGAGTGGGGCAGGTAAGTG
	Reverse	EG_PSMD2_R	TTAGGGTTCTTGCGCAGGAT
	Forward	EG_AP2M1_F	CCTCTCCGAGTCAAACTTGC
	Reverse	EG_AP2M1_R	GCTCTTGTAGAAGCTGGGAGA
CLCN2 qPCR	Forward	CLCN2_qF2	CGGCAGTTTATGCAGGAGAA
	Reverse	CLCN2_qR2	CAATCTGAAAGCGCACTCCT
ALAS qPCR	Forward	ALAS1_qF1	CGAACCTCTCAGAACGGGAA
	Reverse	ALAS1_qR1	CTTGACAGGAATGGGCAACG
PMEL in situ probe	Forward	EG_PMEL_134F	AACAACAGATGGGGGCAGAA
	Reverse	EG_PMEL_867R	GTCCCACTCTGGTCACCAAA
CLCN2 in situ probe	Forward	EG_CLCN2_1579F	CACATCCTGCCGGTTATGA
	Reverse	EG_CLCN2_2209R	ACTCTGCTGTTGAGGCGTTG
CRISPR-Cas9 gene-editing	gRNA	CLCN2_gRNA_578r	CATGCTGGCTATGTGAACAAAGG
	Forward	EG_CLCN2tg-89622-F	TCCAGTTGTCTTTTGAGGGGA
	Reverse	EG_CLCN2tg-90182-R	GTAAAAGGGCCACAGTTGCC
